# The Foundation for Engineering a Pancreatic Islet Niche

**DOI:** 10.3389/fendo.2022.881525

**Published:** 2022-05-04

**Authors:** Smit N. Patel, Clayton E. Mathews, Rachel Chandler, Cherie L. Stabler

**Affiliations:** ^1^ J. Crayton Pruitt Family Department of Biomedical Engineering, University of Florida, Gainesville, FL, United States; ^2^ Department of Pathology, Immunology, and Laboratory Medicine, University of Florida, Gainesville, FL, United States; ^3^ Diabetes Institute, University of Florida, Gainesville, FL, United States

**Keywords:** microphysiological systems, islet-on-a-chip, diabetes modeling, hydrogels, islet, islet physiology

## Abstract

Progress in diabetes research is hindered, in part, by deficiencies in current experimental systems to accurately model human pathophysiology and/or predict clinical outcomes. Engineering human-centric platforms that more closely mimic *in vivo* physiology, however, requires thoughtful and informed design. Summarizing our contemporary understanding of the unique and critical features of the pancreatic islet can inform engineering design criteria. Furthermore, a broad understanding of conventional experimental practices and their current advantages and limitations ensures that new models address key gaps. Improving beyond traditional cell culture, emerging platforms are combining diabetes-relevant cells within three-dimensional niches containing dynamic matrices and controlled fluidic flow. While highly promising, islet-on-a-chip prototypes must evolve their utility, adaptability, and adoptability to ensure broad and reproducible use. Here we propose a roadmap for engineers to craft biorelevant and accessible diabetes models. Concurrently, we seek to inspire biologists to leverage such tools to ask complex and nuanced questions. The progenies of such diabetes models should ultimately enable investigators to translate ambitious research expeditions from benchtop to the clinic.

## 1 Introduction

Diabetes mellitus is considered one of the largest growing healthcare challenges of the 21^st^ century, leaving an ominous global burden on public health. In the past two decades, the number of adults living with this disease has more than tripled and a continuous rise to more than half a billion adults with diabetes by the end of this decade is projected (1). The diagnosis of diabetes occurs when the human body is not able to produce adequate insulin or when cells fail to respond effectively to insulin, causing systemic dysregulation of blood glucose levels. Diabetes can be considered a syndrome that involves many organs/organ systems (*e.g.*, pancreas, liver, adipose tissue, and the immune system). Additionally, health complications can arise from sustained hyperglycemia, including severe damage to the heart, kidney, nerves, vasculature, and retina. This ultimately reduces the life expectancy of individuals with diabetes and imparts global socio-economic impacts. Current treatment options for diabetes range from administration of oral medications to experimental cell-based therapies.

Biomedical research efforts, supported by governmental, philanthropic, and industry sectors, have made significant contributions to our understanding of the physiology and pathologies associated with diabetes; however, major knowledge gaps remain that negatively impact our progress towards a cure. Type 1 diabetes (T1D), for example, is an autoimmune condition where the body is unable to produce sufficient insulin because of a selective autoimmune-mediated attack on the insulin-producing β-cells. Genetics plays a major role in T1D, with over 150 loci linked to T1D susceptibility (2, 3). While genes such as Human Leukocyte Antigen Class II loci are clearly major players in T1D, at the time of writing, the exact role of the genes implicated in T1D pathogenesis has not been fully established. Furthermore, while researchers have gained insight into the peripheral immune and endocrine changes that precede disease diagnosis, the specifics of T1D initiation and subsequent propagation in the pancreas and pancreatic lymph nodes remain undetermined (4). Furthermore, it is unknown if β-cell dysfunction precedes lymphocytic interactions or whether this is a secondary effect. In Type 2 diabetes (T2D), the initiation, progression, and long-term mechanisms are also not well understood (5). People with T2D are challenged in the physiological control of glycemic homeostasis, at least partly due to insulin resistance. In the study of T2D pathophysiology, numerous questions remain, such as the degree of permanent β-cell mass loss, lipotoxicity kinetics in β-cell destruction, and the nuances of the timeline for insulin resistance. Evidently, diabetes is a complex disease initiated and propagated by unknown factors. Gaps in our knowledge delay efforts to find long-standing and effective treatments.

The endocrine organoids at the center of diabetes are the insulin-producing pancreatic islets of Langerhans. Islets are composed of multiple cells type and are responsible for maintaining blood glucose homeostasis. These manufacturing facilities secrete several endocrine hormones necessary for metabolic regulation. Examination of the scientific literature reveals a remarkable understanding of islet micro-architecture, including cellular composition, cell-cell junction physiology, and its surrounding peri-islet extracellular matrix (ECM). Access to such knowledge took decades of research, as methods for the investigation and/or interrogation of islets are limited by material access, species variance (*i.e.*, human vs. other animals) (6), and relevance to native physiology, as well as other factors such as efficiency, cost, and reproducibility. Key obstacles in diabetes research are at least in part due to the insufficiency of current models, such as animals and 2D cultures, in supporting clear experimental control and/or outputs when testing diabetes-relevant hypotheses. Thus, there is a need to engineer additional benchtop platforms that provide further insight into normal and diseased pancreatic tissues.

Designing new solutions to a problem using engineering design processes first demands conducting extensive background research. For biomedical engineering problems, this first starts from understanding the normal anatomy and physiology of the tissue of interest. This foundational knowledge can then be used to identify key design parameters and/or targets. Next, a comprehensive understanding of conventionally available techniques and/or approaches is needed to identify their critical obstacles or deficiencies, as well as costs and market share. From this background knowledge, clear design requirements and engineering criteria for creating prototypes are identified. This review seeks to outline this said engineering process. The first section is dedicated to summarizing the native pancreatic niche, highlighting key unique features. Traditional methods of studying diabetes pathogenesis and potential therapies are also discussed, including their advantages and disadvantages. Subsequently, we summarize alternative strategies that elevate traditional culture methods by employing fluidics and 3D matrices. Lastly, we consider challenges in the adoption, scale-up, and translation of these platforms, as well as key future efforts in modeling the islet niche.

## 2 The Microenvironment of the Pancreatic Islet

### 2.1 General Islet Anatomy

The overall function of the pancreas is to support macronutrient uptake and homeostasis. The adult pancreas can be macroscopically segmented into four major sections: the head, neck, body, and tail. Within the organ, two key architectures are present: the acini and pancreatic ducts that provide the exocrine function to aid digestion in the small bowel; and islets that deliver endocrine function for carbohydrate regulation in the circulation. The majority of the pancreatic tissue is composed of acini, whose secreted digestive enzymes drain into the duodenum of the digestive system. Besides the exocrine tissue, other structures, including blood vessels (arteries, veins, and capillary systems), lymphatic vessels, and nerve fibers (sympathetic and parasympathetic) are also present (7). The interstitial space between each of these components is filled with an ECM. While a pancreas is considered a multi-functional organ, endocrine islets are the key pancreatic tissue of interest as it relates to diabetes.

**Figure 1:**
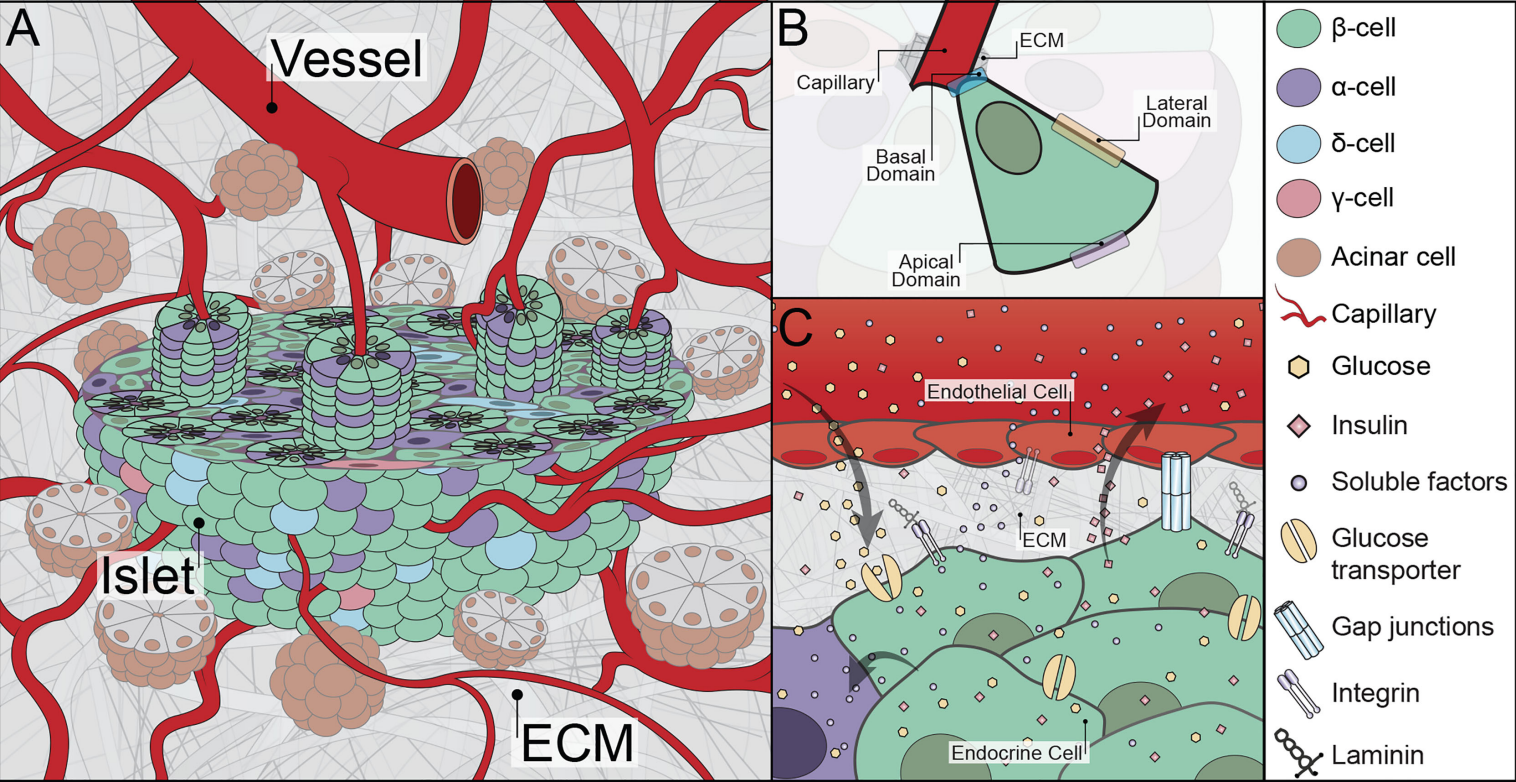
Graphical illustration of relevant islet physiology. **(A)** The native peri-islet space consists of acinar cell clusters surrounding the pancreatic islets. The islets have an afferent blood vessel adjacent to their structure that quickly converts into tortuous capillary systems. A cross-section of an islet highlights the rosette-like architecture composed of endocrine cells wrapped around a capillary. The interstitial space adjacent to the islets also consists of ECM. **(B)** Intra-islet architectural organization in a rosette-like structure where distinct faces of an endocrine cell with respect to the capillary are visualized. **(C)** The subcellular interface of islets showing relevant molecules and cell-cell and cell-matrix interactions. Illustration not drawn to scale.

Endocrine pancreatic islets are dense cellular islands intermingled within the exocrine pancreatic tissue and are easily distinguished by their high vascularization and a distinct layer of ECM coating (**Figure 1A**). Roughly, 1 – 15 million islets (8, 9) are distributed throughout the human pancreas without a clear pattern in terms of regional localization, orientation, and/or size (9, 10). An average islet is 150 µm in diameter with roughly 1500 cells (11), but cluster sizes can vary from as small as tens of microns to as large as half a millimeter in diameter (12). Islet clusters contain a collection of endocrine cells with species-specific differences in their contribution and spatial position. For example, immunohistochemical analysis estimates 60-80% β-cells, 15-20% α-cells, and <10% δ-cells for mouse islets, while human islets contain 50-75% β-cells, 25-35% α-cells, and 10% δ-cells (6, 13–15). Additionally, the mouse β-cells appear to be centrally clustered within the islet, while in human islets β- and α-cells exhibit a less-segregated distribution (15, 16). Cellular compositions can also change with respect to size and anatomical location within the pancreas. For instance, smaller islets contain predominantly β-cells, while larger islets are likely to have other cell types present (17). In rat pancreas, α-cells are found largely in islets located at the tail of a pancreas while γ-cells are predominately found within islets from the head of the pancreas (18, 19). In addition to endocrine cells, islets also contain other cell types (*e.g.*, vascular, mesenchymal stromal, immune, and neural cells; see **Table 1**). Globally, the variances in islet composition due to species, size, and location should be taken into consideration when conducting *in vitro* analysis of islet physiology.

**Table 1:** Current understanding of different cells residing within the pancreatic islets. [Adapted from (17, 18)].

Cell Types within the Pancreatic Islet	
**Endocrine cells**	α-, β-, δ-, γ [pancreatic polypeptide (PP)]-, ϵ-, ghrelin-cells
**Vascular cells**	Endothelial cells (ECs), pericytes, vascular smooth muscle cells
**Mesenchymal stromal cells**	Fibroblasts, myofibroblasts, adipocytes, duct cells
**Immune cells**	Granulocytes, lymphocytes, resident macrophages, dendritic cells, mast cells
**Neural cells**	Neurons, Schwann cells

In addition to cellular composition, the features of the ECM surrounding and within pancreatic islets are notable. Islet ECM is found within two distinct locations: 1) peri-islet ECM, which separates the intra-islet cells from the exocrine tissue; and 2) vascular ECM, which encases the intra-islet capillaries and serves as the interface between endocrine and endothelial cells (ECs) (see **Table 2**). The peri-islet ECM is comprised of both a basement membrane (BM) and an interstitial membrane (IM) ECM. The peri-islet BM is mainly comprised of collagen IV and laminins, with collagen providing structural integrity and laminin imparting biological activity and cell signaling (16, 21, 28). Other ECM molecules are also present and contribute to its organization and function (**Table 2**) (16, 21, 27). For example, collagen and laminin self-assemble with either nidogen or perlecan to create a tight network (23). ECM subtypes within the peri-islet BM can be species-dependent. For example, laminin isoforms shift from LM-411 and LM-211 in mice to LM-511 and LM-211 in humans (20, 21, 23). Surrounding the peri-islet BM is the peri-islet IM, which is a looser network of proteins composed primarily of fibrillar collagens (*i.e.*, col-I, col-III) with other matrix molecules (**Table 2**) (21, 24, 29).

**Table 2:** Currently identified major ECM types in adult pancreatic islets. Species-specific information is included in the table when appropriate with literature references.

Peri-Islet ECM		Vascular Intra-Islet ECM
**Peri-islet BM**	**Peri-Islet IM**	**Vascular BM (mice) and Vascular BM + Peri-islet BM (humans)**
Collagen-IV [α1 & α2 in mouse islets (20)]	Collagen-I, -III, -VI (21)	Collagen-III, -IV, -VI (16, 21, 22)
Laminins [LM-211, -411 in mouse islets (21); LM-511, -521 in human islets (23)]	Fibrillin-2 (21)	Laminins (LM-211, -221, -411, -421, -511, -521 in human & mouse islets) (24–26)
	Tenascins (21)	
Heparan Sulfate Proglycans (*i.e.*, Perlecan) (21)	Matrilin-2 (24)	
	Chondroitin (21)	
	Dermatan Sulfate Proteoglycans (21)	
Nidogen-1, -2 (21)	Vitronectin (21)	
Fibronectin (27)	Fibronectin (21)	

Within the islet, ECM appears to be exclusively relegated to the outer coating of intra-islet capillaries (25). In mice, only a single layer of vascular BM is detected. In humans, two BM layers are present, with the outer layer arising from the invagination of the peri-islet BM. The major ECM components of the intra-islet ECM are collagens and laminins (16, 21, 22, 26) (**Table 2**). For human islets, the ECM components on either side of the double membrane vary. For example, the laminin composition interacting with endocrine cells is dominated by LM-511, while the side facing endothelial cells is a composite of LM-411, -421, -511, and -521 (26).

There is a growing interest in understanding the cells primarily responsible for secreting ECM molecules within and around the pancreatic islet. Evidence suggests that ECM is secreted by ECs and not β-cells (25, 30–32). However, other vascular cells, mesenchymal stromal cells, or/and even neural cells (*i.e.*, pericytes, fibroblasts, myofibroblasts, or Schwann cells) may contribute to ECM production (21, 33).

It is well established that the interaction of islets with the ECM can regulate cell survival (34–37), proliferation (16, 38), and insulin secretion (39–41). The prompt loss of peri-islet ECM, *e.g.*, collagen IV, laminin, and perlecan, during the enzymatic digestion process of islet isolation leads to the induction of islet cytotoxicity and dysfunction (42). A correlation between the islet ECM and T1D has also been reported, where T1D pathogenesis is associated with both alterations in ECM composition and autoreactive T cell-mediated ECM degradation (21, 43). Overall, when designing systems for studying pancreatic islets, special care should be given to the preservation of its unique anatomical, cellular, and ECM components.

### 2.2 Pancreatic Islet Vascularization

Within the pancreas, islets are distinguished by their robust vascular network, which plays a key role in both nutritional support and function. Vascular perfusion of the islet is remarkable; globally islets receive up to 23% of the overall pancreatic blood flow despite comprising only 1–2% of an entire pancreatic mass (44–47). Studies in rat islets estimate an average islet blood flow rate of 69 µl/min/pancreas, which increases up to 125 µl/min/pancreas in response to elevated blood glucose (47, 48). Copious islet perfusion serves to provide optimal glucose sensing to facilitate insulin or glucagon release, as blood exiting the islet flows through the branches of the descending aorta and drains to the portal vein (49, 50). High blood flow capacity also supports the substantial metabolic demands of the islet (51, 52).

Beyond arterial and venous blood flow, interstitial flow between the individual cells of an islet further facilitates nutrient exchange and paracrine signaling. The interstitial flow velocity of physiological tissues is generally postulated to range from 0.1 – 2 µm/s (53); however, islet-specific interstitial flow parameters have not been directly measured. From a fluid mechanics perspective, the shear stress experienced by individual islet cells within their niche is expected to be orders of magnitude lower than the shear stress exerted on the ECs lining the vascular conduits (*i.e.*, arterioles and venules). Unlike the rest of the pancreas, however, islets may be largely deficient in lymphatic capillaries (54, 55). This could play a significant role in altering theoretical shear stresses and pressures within the islet interstitial space, particularly during a high glucose challenge.

The architectural design and cellular organization of the vascularized islet are distinctive. The afferent vascular arterioles entering an islet are converted to a dense and highly fenestrated network of capillaries (**Figure 1A**) (56). Capillaries are comprised of an inner layer of endothelial cells and an outer coating of pericytes and vascular ECM (57). These micro-vessels have 20-30% larger diameter than those in exocrine pancreatic tissue and are intertwined between the endocrine cells (18, 58). In rats, β-cells are reported to always be adjacent to at least one capillary ECs (59). In humans, this is less dogmatic, as intra-islet capillaries are primarily surrounded by α-cells (60). Human β-cells can be found two or even three cell layers away from a capillary (61); however, the connection between human β-cells and ECs is dynamic, with some β-cells found extending between α-cells to reach the outer capillary BM (61). The intimate interface of endocrine and endothelial cells is architecturally accomplished by their organization in a rosette-like structure around the blood capillary (**Figure 1B**) (17, 59, 62). In addition, another set of capillaries follows a tortuous path around the periphery of the rosette-like structure (59). It is presumed that the capillaries surrounding the rosette are arterial capillaries delivering metabolites, while the capillaries at the central lumen of the rosette are venous capillaries transporting waste away from the cells (59, 62); however, further definitive evaluation is required.

### 2.3 Distinct Islet Subcellular Interfaces

Intra-islet subcellular interfaces contain cell-cell junction proteins, transport channels, soluble factors, and ECM molecules that coordinate individual cellular responses into collective islet function (**Figure 1C**). For example, β-cells synchronize Ca^2+^ oscillations to efficiently initiate and promptly stop insulin secretion. Within the rosette structure surrounding the intra-islet capillaries, endocrine cells are delegated into three distinct faces, with a corresponding physiology that reflects their architectural design: i) the edge near the ECs (the basal domain), ii) the membrane laterally connecting to neighboring endocrine cells (the lateral domain), and iii) the edge furthest away from the ECs (the apical domain) (**Figure 1B**) (63). The basal domain of the cell is unique in that it directly interacts with the vascular ECM. As such, cellular adhesion molecules are largely ECM binding receptors, such as integrin β1 (23, 25, 64). It has been proposed that the majority of insulin granule fusion and release is targeted at the basal domain (65, 66). Furthermore, published reports indicate a link between the engagement of integrin β1 and elevated β-cell proliferation and function (26, 64, 67). Moving away from the capillary and into the lateral domain, cell surface proteins shift to cadherins (*i.e.*, E-Cadherin), connexins (*i.e.*, Cx36, Cx30.2), and ephrin complexes (*i.e.*, ephrin-Eph), which are more common in cell-cell interactions (63, 68, 69). Both cadherins and ephrins play critical roles in the preservation of β-cell function. as disruption of these interactions, *e.g.*, β-cell dissociation, leads to loss and/or dysfunction in glucose-stimulated insulin secretion (69–71). Connexins, transmembrane proteins that form gap junction channels and provide intracellular communications, are also found within discreet locations in the pancreatic islet (68). Finally, the apical domain consists of primary cilia (*i.e.*, acetylated tubulin) and tight junction proteins (*i.e.*, ZO-1, PAR-3) (63). Select reports implicate cilia dysfunction in T2D development (72). Altogether, it is clear that the subcellular interactions within the islet are not random and appear coordinated around the capillary network.

The interface of β-cells with other endocrine cells contains transport channels that passively or actively facilitate the diffusion of key molecules such as glucose, calcium, and sodium to support insulin secretion (**Figure 1C**). Classically known glucose transporters play key roles in the passive diffusion of glucose. Moreover, hormones released by non-β endocrine cells not only contribute to whole-body glycemic regulation (*e.g.*, pancreatic polypeptide regulates food intake and glucagon directs liver-facilitated glucose release) but also provide local regulation of islet responses (*e.g.*, somatostatin release by δ-cells inhibits insulin and glucagon secretion in β- and α-cells, respectively). Additional non-hormonal soluble agents (*e.g.*, hepatocyte growth factors, angiogenic growth factor, and endothelin-1) are also found at the islet subcellular interface and contribute to cellular regulation (68, 73).

Gaining a clear understanding of pancreatic islet anatomy and physiology, ranging from the macroscopic niche to subcellular interfaces, provides a template for identifying key parameters that are important to preserve or mimic *in vitro*. As islets are complex micro-organs that are impacted by significant species-dependent heterogeneity in general anatomy, there is no such thing as a stereotypical islet. Within the native islet niche, islet cells are embedded within ECM that serves to anchor the tissues into 3D space for cellular migration and remodeling. Islet vasculature is key in supporting the metabolic demands of the cells and for optimal sensing and release of endocrine hormones. Lastly, subcellular features within islets and their polarity for specific interactions with respect to the vasculature provide critical cues in supporting islet functionality. When engineering the islet niche, these complexities will create difficulties in the generation of a wholly physiological system; however, hypothesis testing can allow for their refinement in assembling specific features to inform and/or predict *in vivo* outcomes.

## 3 How to Study Diabetes?

**Figure 2:**
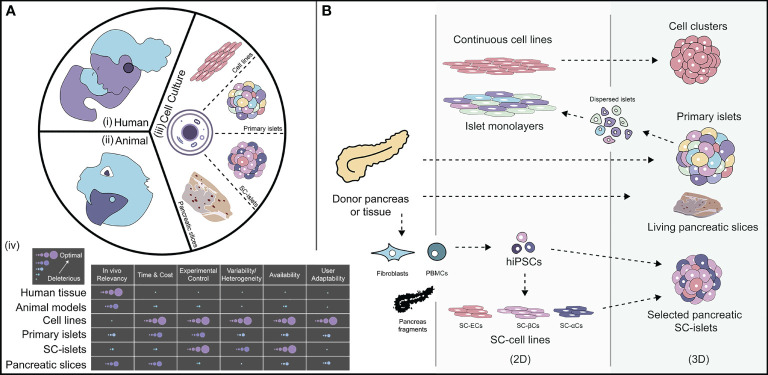
The landscape of how diabetes is studied and cell sources available for researchers. [**A** (i-iii)]: Currently available models to study diabetes, such as human clinical trials, animal pre-clinical models, and *in vitro* cell culture systems. [**A**-(iv)] Relative breakdown of different characteristics of each available model for diabetes to highlight key advantages and disadvantages. **(B)** An outlook of available cell sources for diabetes modeling separated by 2D and 3D morphology. PBMCs, peripheral blood mononuclear cells; hiPSCs, human induced pluripotent stem cells; SC, Stem cell-derived; ECs, endothelial cells; βCs, β-like-cells; αCs, α-like-cells. Illustration not drawn to scale.

The study of any disease generally starts with models as simple as cells in test tubes, which progress in complexity through small and large animal models and ultimately to the clinical setting with human subjects. Here, we outline key assets available in the study of pancreatic islet physiology and pathology (**Figure 2A**). We also provide historical perspectives, when relevant, and denote the advantages and disadvantages of each model.

### 3.1 Acquisition and Study of Human Samples

Within the United States, both government and private foundations have established resources for the distribution of human pancreatic islets procured from brain-dead organ donors [**Figure 2A**(i)]. The National Islet Cell Resource Center (ICR) consortium, founded in 2001, is the largest organized effort in the world and globally provides human islets for research (74). Since 2009, ICR operates as the Integrated Islet Distribution Program (IIDP), which is currently funded by The National Institute of Diabetes and Digestive and Kidney Diseases (NIDDK) (75). The IIDP utilizes five U.S.-based certified islet isolation centers for the procurement of islets from cadaveric organ donors where, as of 2022, they have cumulatively made nearly 15,000 shipments of islets to researchers (76). Since 2016, participating investigators who receive human islets have access to The Human Pancreas Analysis Program (HPAP) database (77). Anonymized information available includes, clinical and donor islet phenotypic data (*e.g.*, pre-shipment islet preparation information, histology, mass cytometry, oxygen consumption, functional perifusion, calcium imaging, and electrophysiological measurements), as well as epigenomic data (*e.g.*, islet cell sequencing data and immune cell characterization), to better contextualize donor characteristics. The HPAP database is further sectioned into three branches: i) Pancreatlas (78), ii) The Human Islet Phenotyping Program (HIPP) (79), and iii) Diabetes Epigenome Atlas or The Human Islet Genotyping Initiative (HIGII) (80). An additional resource is The Human Protein Atlas, which provides proteomics information of the human pancreas and other tissues (81). Together, these portals serve as detailed and comprehensive resources of information regarding human islets procured from non-diabetic and diabetic donors (*i.e.*, T1D, T2D, and monogenic forms of diabetes). Moreover, the Network of Pancreatic Organ Donors with Diabetes (nPOD) biobank provides live and fixed samples from the pancreas, spleen, lymph node (including pancreatic lymph node), thymus, and bone marrow from cadaveric organ donors, with, without, or at-risk of developing T1D (82). In addition, there are commercial-based sources of human islets and T1D-relevant tissues [*e.g.*, Prodo Laboratories (83)]. Most importantly, access to these precious materials and the scientific knowledge held within is only possible due to altruistic donations. The scientific community expresses our highest gratitude to these donors and their families.

Studying diabetes using human samples can provide the most relevant information in regards to a human disease. However, the total number of donors is limited in terms of procurement, costs, consistency of product, and capacity for durable interrogation [**Figure 2A** (iv)]. The primary source of human pancreatic tissue for pathophysiological studies is deceased donors, which become available at discreet and unpredictable time points. While biopsies from non-terminal patients undergoing partial pancreatectomy are occasionally available (84), this is an unreliable option for non-diseased or damaged pancreata. The elective procurement of pancreatic biopsies from otherwise healthy T1D individuals is problematic, as illustrated by the Diabetes Virus Detection Study (DiViD), which was terminated due to serious complications (85). Overall, mechanistic temporal studies of diabetes pathogenesis using living human models are limited to peripheral blood cells and proteins. Thus, there is a significant limitation in the type of queries that can be made using human subjects, as well as the supply and reliability of human T1D tissues (86).

### 3.2 Animal Models

Animal models, including their explanted cells and tissues, have been used in basic science to study or understand biological phenomenon since the fourth century before the Common Era [**Figure 2A** (ii)]. In diabetes research, animal models have provided major discoveries resulting in clinical advances in the treatment of diabetes, as well as a basic understanding of disease initiation and propagation factors. Diabetes can be studied in animal models using either strains that spontaneously develop diabetes or genetic- or virally-induced models (87, 88). The most common strains for spontaneous T1D are the non-obese diabetic (NOD) mouse and the BioBreeding Diabetes-Prone (BB-DP) rat models (88, 89). These two strains, and their derivatives, exhibit genetic and environmental features that are relevant to human T1D, such as the essential role of specific MHC alleles and the emergence of β-cell-specific autoimmune antibodies against insulin, glutamic acid decarboxylase (GAD), and islet cell antigen 512 (IA-2), as well as autoreactive helper (CD4^+^) and cytotoxic (CD8^+^) T cells that recognize β-cell antigens similar to the human condition (90). The induction of T2D involves using obese and non-obese models that render insulin resistance and/or β-cell failure (87). Some commonly used spontaneously developed T2D models are strains carrying *Lepr* ^db/db^, Kuo Kondo (KK)-, and New Zealand obese (NZO)-mice, which exhibit hyperglycemic symptoms and develop insulin resistance (87, 91). Diet-induced obesity is routinely used in mice, rats, and hamsters (*e.g.*, Israeli sand rats); however, there are challenges with human relevancy with respect to diet and lifestyle. Alternatively, insulin-dependent diabetes can be induced *via* β-cell removal through chemical targeting and destruction with streptozotocin (STZ) or alloxan (AL), or through complete pancreatectomy (90, 92). Injurious β-cell killing or removal provides the advantage of inducing a predictable and overt diabetic state within any animal, which is useful for examining the efficacy of cell therapies and/or the study of long-term complications arising from hyperglycemia. However, this approach is not appropriate for the interrogation of autoimmune-related immunological pathways or for the study of β-cell dysfunction.

While not fully representative of human pathophysiology, the value of using animal models is their capacity to invasively investigate complex biological pathways that are not feasible in human subjects. Various mouse strains permit the interrogation of genetic risk, environmental cues, and therapeutic interventions within a model supportive of temporal analysis and enhanced experimental control [**Figure 2A** (iv)]. It also delivers a full spectrum of *in vivo* cues that are impossible to recapitulate *in vitro*. Furthermore, as imaging modality capacities are enhanced, unprecedented insight into temporal and *in situ* phenomenon is now feasible (93, 94). Research using T1D-relevant murine strains has resulted in significant contributions in the understanding of insulitis, β-cell stress, and islet-relevant immunological responses, as well as in the optimization of cell therapy for the treatment of T1D (89, 95). Further advancements in the genetic engineering of murine strains, and in efforts to generate humanized mouse models, will enhance their utility and relevancy (96). Finally, the validation of the safety of therapeutic approaches in small and large animal models is typically necessary in the regulatory approval of clinical trials.

The use of animals, from their cells and tissue explants to whole organism models, for understanding human-based phenomenon has its limitations. Animal models possess key phenotypic and genotypic differences, along with variances in anatomy and physiology, metabolism, and immune responses. Cell distribution, composition, and function vary with respect to the animal species, which challenges the general extrapolation of results to humans (97, 98). Glycemic control, processing, and diet also play a role in the clear interpretation of impact (92). Furthermore, experimental handling challenges should be considered. Different cohorts of the same strain of mice exhibit variation in basal glucose levels, the sensitivity of STZ, insulin secretion abilities, and sex differences, while the immunological maturity of mice and rats bred within pathogen-free facilities is depressed (92, 99). Thus, care should be taken in designing an interventional study and interpreting its results. Finally, the detailed interrogation of cells and tissues within the living animal is limited. This has motivated the use of *in vitro* cellular models, which provide a different perspective in studying diabetes.

### 3.3 Cell Culture Models | Cell Source

A wide range of cells and tissue subtypes are grown in laboratory incubators, from two-dimensional (2D) monolayer cultures to complex multicellular 3D tissues. These biologic sources generally exhibit advantages of simplicity, low cost, reduced variability, ease in manipulation, and robustness in high throughput screening [**Figure 2A** (iii)]; however, the simple culture of isolated cells and tissues within basic cell culture flasks or dishes is insufficient to fully and durably support native cellular phenotype. Herein, we discuss categories of cell and tissue sources available for diabetes research and globally highlight their benefits and pitfalls (**Figure 2B**).

### 3.4 Continuous Cell Lines

As β-cells play a predominant role in the development of both forms of diabetes, numerous β-cell lines that mimic various features of glucose-responsive insulin secretion have been established and/or engineered (100, 101). In general, most cell lines are tumor-derived or generated from the incorporation of viruses into fetal, neonatal, or adult pancreatic fragments (102). The advantages of these continuous β-cell lines are ease in culture and expansion, making them a convenient source for screening and testing new approaches or for the interrogation of distinct pathways (102). β-cell lines can be cultured as either 2D monolayers or 3D organoids, providing flexibility in culture formats. Many also exhibit sensitivity to immune-mediated cell death (102). Results from these cells, however, must be contextualized by their limitations. For example, the kinetics of insulin secretion and glucose sensing in continuous β-cells are generally non-physiological, as most exhibit either hypersensitive or insensitive insulin kinetics to glucose challenges. Their oncogenic nature also requires care in the direct extrapolation of results exploring β-cell stresses or cytotoxicity screens. Additionally, β-cell lines lack multi-cellularity and ECM, which likely contribute to native β-cell responses. Efforts to enhance cell line models include the integration of other cell types into the 2D monolayers or 3D organoids (103–105) and the culture of cells on ECM coated surfaces (64, 106). While continuous β-cell lines are highly useful and efficient for testing and screening, their lack of native human islet features ultimately requires results to be confirmed using primary pancreas tissues.

### 3.5 Primary Islets

Our present knowledge of β-cell pathophysiology has mostly stemmed from the interrogation of isolated pancreatic islets. The possibility of isolating islets was first suggested as early as 1911 (107), but the isolation process was not fully optimized and automated until closer to the late 1980s (108–111). While the primary β-cell is of main interest to diabetes researchers, β-cell function is better retained when cells are kept within the 3D primary islet structure. Primary pancreatic islets are typically cultured in static systems on non-adherent plates, in order to retain their multicellular, 3D architecture. The dispersion of primary islets and subsequent culture of primary β-cells in 2D monolayers results in dysfunction of insulin secretion, likely due to loss of key cell-cell interactions (69–71). Although, the culture of dispersed islet monolayers onto defined surfaces can provide single-cell resolution of key subcellular interactions (112). Islets can also be dispersed, sorted and/or virally transduced, and reassembled, which provides insight into the roles of cell-cell and/or cell-matrix interactions (113); however, this process typically leads to significant cell loss. In general, isolated islets provide intimate investigation and unique insight into diabetes-related hypotheses *via* knock-down studies, genetic manipulations, or procurement from donors at specific disease states.

The utilization of primary pancreatic islets to address questions related to native or disease physiology, however, is limited by the deleterious pathways activated at the start of the islet isolation process. Specifically, isolated islets quickly exhibit a loss in islet structural integrity (114), up-regulation of stress genes and cytokines (115, 116), increased reactive oxygen species (ROS) production (117), and activation of inflammatory (118) and apoptotic pathways (119). Islet decline is unresolved in culture, as the lack of the ECM niche and ideal culture conditions creates anoikis-initiated apoptosis and insulin dysfunction (120, 121). Thus, the utility of primary islets is extremely limited by temporal constraints, with most islet studies conducted within less than one week post-isolation. The accessibility of primary human islets is also limited to the availability of donor tissues. While animal tissues are more easily procured, key genotypic and phenotypic mismatches must be considered when extrapolating results. Overall, the utilization of primary islets can be a powerful resource in the pipeline of tools to understand native physiology and disease progression, but significant improvement in culture parameters is necessary to facilitate stable long-term islet culture.

### 3.6 Stem Cell-Derived Islet Organoids

A promising approach to enhance both the access and relevance of cell cultures to human physiology is the development of stem cell-derived organoids, which can theoretically provide an infinite supply of cells. Human endocrine and non-endocrine cells can be generated from both human embryonic stem cells (hESCs) and human-induced pluripotent stem cells (hiPSCs), which are typically procured from living donors (**Figure 2B**) (95, 122). Stem cell-derived β-like-cells (SC-βCs) created from earlier differentiation protocols were insufficiently mature *in vitro* and required animal implantation to elevate their functionality (123–125). More recent SC-βCs differentiation protocols have shown improved *in vitro* maturation with increased insulin secretion to glucose challenges (*e.g.*, exhibition of both first and second phases of insulin secretion) and functionally mature mitochondria (126–131). As advances are made in recreating islet-like organoids *in vitro*, unique research questions can be explored. For example, one can examine patient- and disease-specific differences from their respectively derived SC-βCs (132). Cells can be genetically manipulated to enhance cell fate mapping and impart distinct risk or protective alleles. Moreover, other islet-resident cells can be incorporated, such as ECs, α-cells, and δ-cells, to mimic the native islet environment (122). The potential to completely customize the final stem cell-derived islet organoid provides unprecedented control to experimental studies.

Limitations of these cells still exist, as they lack full functional maturity (133). For example, key β-cell transcription factors are decreased in SC-βCs, when compared to isolated islets (134). While some differentiated protocols exhibit biphasic insulin secretion profiles, the magnitude of the second phase insulin secretion is still much lower than native islets, which may lead to insufficient efficiency in blood glucose clearance. From the perspective of scalability and manufacturing, there are still challenges with limited shelf-life and inter- and intra-batch reproducibility of the final product. Efforts in creating cell culture methods that more closely recapitulate key features of the native *in vivo* environment (*e.g.*, oxygen gradients and ECM niche), as well as efforts that support their long-term culture to enhance maturity, may serve to resolve these issues (41, 135). Overall, advancements in islet stem cell biology create a promising avenue for researchers to incorporate these cells within their proposed disease models.

### 3.7 Living Pancreatic Slices

Given the challenges in islet isolation and SC-βCs differentiation, the emergence of organ pancreatic slices provides a distinct *in vitro* tool, where islets are retained within their 3D pancreatic niche and cultured as a pancreatic tissue segment. Inspired by brain tissue slicing techniques, pancreatic slices are created by encasing the organ in agarose and sectioning 100 – 200 µm thick slices using a vibratome (136, 137). Slices are then typically cultured free-floating in media. With this approach, the islets and their peri-islet niche, including ECM, vasculature, lymphocyte, and neural cells are generally preserved, permitting *in situ* assessments of not just endocrine and exocrine cells but also of the additional contributing peripheral cells. Common readouts obtained from these slices are dynamic live imaging confocal microscopy of the identified islets within the slices, indirect measurements of hormones released in perifusion experiments (138), and/or electrophysiological characterization using patch-clamp technology (139). Numerous queries can be made across the translational spectrum by using pancreas slices from animal and human donors both in healthy and diabetic states. For example, T1D pathogenesis was analyzed using slices from human pancreatic donors by showing loss of β-cells and immune cell infiltration (140). T2D human slices were utilized to show that β-cell dysfunction transpired even when β-cell mass was intact, providing evidence of features exhibited during early T2D pathogenesis (84). Additional emerging research is using these slices to explore diabetes pathogenesis and the islet-immune interface (138).

While this approach serves as a great platform, slices are challenged by their inherent complexity and restricted access, particularly from human source. The heterogeneity of human donors is extremely high, as the slices are obtained from 0.5 cm (3) blocks and are procured from cadaveric donors. Slices are delicate and their durability in culture under traditional static conditions is typically limited to only a few days, although more advanced culture platforms, such as oxygen-enhanced transwell systems and the use of protease inhibitors, indicate the potential to extend their culture (141). Enhancing the duration of culture will open up opportunities for more advanced temporal multi-omics studies. The unpredictable distribution and size of the islets impart heterogeneity and bias of these studies, as slices rich in pancreatic islets are typically selected for study and larger islets are challenging to examine due to slice thickness. The impact of the mechanical slice on activation of stress factors within the cells is also unknown at this time. Finally, in the study of T1D pathophysiology, care must be taken in the contextualization of observed immunological processes, as cells and pathways identified within these slices at the time of examination are biased by the duration of disease. In general, living pancreatic slices have the potential to subjugate diabetes research efforts, as this relatively new sample type provides a unique *in vitro* window into the pancreatic islet niche.

To conclude, diabetes research has typically relied on conventional human, animal, and cell culture models; however, these cells and tissues exhibit unique features and demands that must be considered prior to the extrapolation of results. To select a specific source for study, one must consider and balance various parameters such as *in vivo* relevancy, time, cost, manipulability, tissue availability, and user adaptability [**Figure 2A**(iv)]. Species source is also a critical choice. While human models are likely the most relevant, access and manipulation are extremely limited. Murine models are comparatively easy to obtain and are more supportive of customization; however, they are restricted in their translatability to mimic a human disease. SC-βCs are flexible for manipulation and can be generated from heterogeneous donor sources, but suffer from complexities in differentiation and may lack consistency and maturity. Finally, pancreatic slices exhibit the advantages of *in situ* study, but elevate the challenges of stable culture. Overall, not one viable source provides the perfect model for studying T1D and multiple cell sources should be used and compared to elevate *in vitro* diabetes research and disease modeling. Furthermore, to elevate the physiological relevance of these cells, additional culture strategies for more closely mimicking the unique islet niche should be integrated.

## 4 Strategies to Engineer an Islet Niche

When examining the breadth of current diabetes disease models on a simple grid of relevancy versus time and cost, it is obvious that standard culture models and whole organism animal models fall into two extreme locations. Traditional cell cultures are highly simplistic in their static 2D environment, while the complexity of animals impairs full control and interrogation capacity. This large gap in biological platforms supports the critical need and the value proposition to engineer more efficient and effective disease models. To provide clear value, engineered models must not sacrifice biological relevancy; concurrently, they must accelerate the scientific discovery timeline while being economically viable. Building these *in vitro* engineered islet models also demands a clear identification of what key aspects of the native peri-islet space are crucial to mimic. Given that the two key features needed for recreating an islet-supportive niche are a 3D microenvironment and dynamic perifusion, subsequent sections highlight the integration of 3D hydrogel systems and the utilization of suitable microphysiological systems (MPS). We then explore how these systems can be customized to provide distinct insight into diabetes-relevant hypotheses.

### 4.1 3D Hydrogels

**Figure 3:**
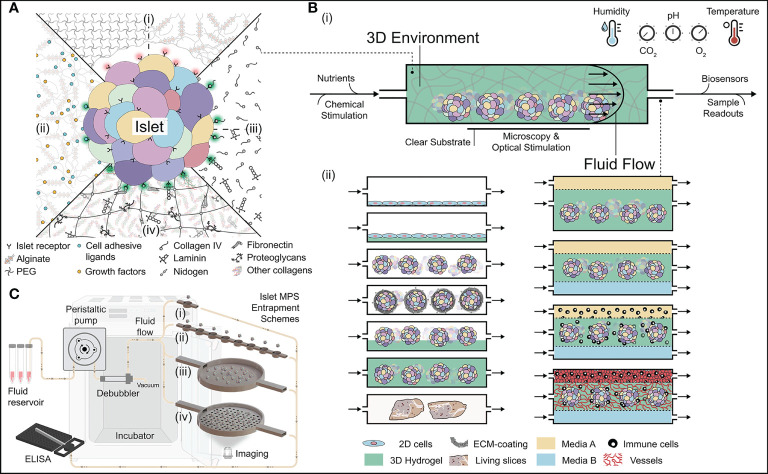
Approaches in building the next generation of 3D islet niche. **(A)** Schematic of different types of 3D hydrogels for culturing islets. [**A**-(i)] Bioinert hydrogels that are naturally non-adhesive to islets. [**A**-(ii)] Functionalized bioinert hydrogels with the incorporation of cell adhesion ligands and growth factors. [**A**-(iii)] Hydrogels with soluble ECM. They could be made either using singular or complementary sets of ECM molecules. [**A**-(iv)] Multi-component decellularized 3D hydrogels with extensive adhesion capabilities to the islets. **(B)** Fundamental features of islet microphysiological systems (MPS). [**B**-(i)] Housing islets within a 3D space require the orchestration of several parameters. [**B**-(ii)] Various MPS schemes for housing islets with hydrogels and other cells/tissue types. [**C** (i-iv)] Different types of islet entrapment schemes using architectural design. Various accessories needed for culturing islets within the MPS are also visualized. Illustration not drawn to scale.

Hydrogels are 3D structures formed from physically or chemically cross-linked polymers that retain large amounts of water (typically > 90% v/v). While these hydrating gels are more commonly employed in T1D for pre-clinical and clinical cell therapy approaches (142, 143), here we discuss the utility of 3D hydrogels for use within an *in vitro* islet niche. Hydrogels employed for islet support can be globally sorted into four main categories: i) bio-inert hydrogels; ii) functionalized bio-inert hydrogels; iii) hydrogels made from discreet soluble ECM components; and iv) decellularized ECM (dECM) hydrogels (**Figure 3A**). When used as islet niches, these matrices can support 3D distribution, mechanical protection, and cell-matrix interactions. In addition, they can support the generation of more complex 3D structures, such as vascular networks.

#### 4.1.1 Bio-Inert Hydrogels

Bio-inert hydrogels lack natural interactions with cells, thus they are typically non-adherent. These 3D hydrogels are made by cross-linking either synthetic or naturally derived materials [**Figure 3A** (i)]. Bio-inert hydrogels offer the advantages of minimal immunogenicity and protein absorption, decreased cell degradation and/or remodeling, and control over degradation profiles and release kinetics (144, 145). Hydrogels made from synthetic polymers typically exhibit stability and reproducibility, as well as more tunable control of viscoelastic and mechanical properties to mimic tissue-like characteristics. Commonly used 3D synthetic hydrogels for encapsulation of islets are polyethylene glycol (PEG) (143) and poly(vinyl alcohol) (PVA) (146). Alternatives to synthetic-sourced materials are naturally derived polysaccharide bio-inert hydrogels, such as alginate and agarose hydrogels. The low toxicity, ease of gelation, mechanical stability, and transparency of these materials make them appealing for islet-based applications, particularly for cellular encapsulation (142, 147–149). Both alginate and agarose can support islet culture (150–155) and permit islet immobilization for cell-specific spatiotemporal tracking (156). The use of bio-inert hydrogels can be challenged by their non-adhesive and inert nature. It can induce apoptotic pathways which can be further exacerbated during long-term islet culture (145, 157). Collectively, the ease of stability of bio-inert hydrogels provides a robust option for creating 3D islet micro-niche.

#### 4.1.2 Functionalized Bio-Inert Hydrogels

Bio-inert hydrogels can be customized to impart specific biological features, such as the incorporation of growth factors, enzyme-sensitive peptide sequences, and/or discreet cell attachment sites to enhance cell proliferation and function [**Figure 3A** (ii)] (158). The incorporation of specific features into synthetic hydrogels has improved islet function, stability, and viability *in vitro*. For example, the functionalization of PEG with arginyl-glycyl-aspartic acid (RGD) peptide or with collagen IV and laminin increased islet insulin secretion (159, 160), whereas the conjugation of PEG with vascular endothelial growth factor (VEGF) promoted graft vascularization (144). While a bit more arduous than synthetic polymers, alginate can also be functionalized to provide various ECM components to improve cell survival and reduce cell apoptosis (161, 162). Adding collagen to alginate hydrogel may further support islet functionality by providing elevated cell-matrix interactions (163). While the selection of key bio-active features and their stable integration into the material can be a challenge, these materials can provide discrete control over the customization of the hydrogel, supporting the clear investigation of a specific material parameter on islet culture.

#### 4.1.3 Discreet Soluble ECM Hydrogels

Singular or combinatory soluble ECM hydrogels is another approach for improving islet culture, as pancreatic islets are naturally embedded in a peri-islet ECM comprised of both basement and interstitial matrix proteins [**Figure 3A** (iii)]. Leveraging what is known about the native peri-islet ECM, hydrogels made of soluble collagen I, III, and IV have been created that exhibited preserved function and suppression of cell death (164–166). A drawback of integrating distinct and soluble ECM proteins into bulk hydrogels is that their resulting conformations and interactions with the encapsulated islets are likely non-physiological. For instance, collagen IV chains are naturally formed in the presence of laminins and nidogens (167); thus, collagen-only hydrogels may lack its native 3D chain configuration. Fibrin is another ECM-based hydrogel that can maintain islet viability and function *in vitro (* 34, 168–170). As an easily cell-degradable material, fibrin may also be suitable for providing a dynamic environment for islets to remodel within a 3D space (171). In addition, fibrin is a common material used to support the formation of *in vitro* vascular networks, which could be combined with islets to form vascular structures around these spheriods (172, 173). Combinatory ECM-based hydrogels can also be made to provide further customization (*e.g.*, hyaluronic acid and collagen hydrogels) (174). While reports on the survival (36), insulin gene expression (25), and morphological changes (112) of β-cells are varied and subjugated by the diversity of ECM molecules, the use of soluble ECM-based hydrogels can nonetheless create a distinct islet niche that not only support islets, but provides dynamic flexibility to support other cell types and/or tissue structures.

#### 4.1.4 Decellularized ECM Hydrogels

Multi-component ECM, sourced from decellularized organs, is another hydrogel option for islet culture [**Figure 3A** (iv)] (29, 175, 176). In contrast to artificially combining distinct ECM proteins into a hydrogel, dECM naturally contains a diverse spectrum of protein molecules to provide embedded cells with physiological signals from multiple proteins, peptides, and factors. While some groups utilize the 3D decellularized structure to seed cells and serve as a conduit for implantation (177), an alternative approach is to grind the dECM into a fine powder. Rehydration of dECM powder results in a self-assembled and nano-fibrous 3D dECM hydrogel with customized mechanical and biochemical parameters *via* modulation of matrix concentration, decellularization process, tissue source, and composition (41, 178, 179). For example, the concentration of the matrix can be modified to provide distinct viscoelastic properties and even mimic selected mechanical features of the native pancreas (41).

The encapsulation of human and rat islets within the dECM hydrogels, sourced from porcine lung, bladder, or pancreas, retained islet functionality in culture and altered the secretion of cytokines and metabolic hormones, when compared to standard 2D static culture (41). Others observed correlations between ECM composition and the functionality of β-cells and porcine islets (178, 179). For stem cell-differentiation towards the β-cell fate, the co-culture of pancreatic progenitors with dECM appears to improve their β-cell maturation (180, 181), although a recent report indicates that this is likely driven by collagen V (182).

Key challenges in the use of this material relate to the processing method, which can vary depending on the tissue type, the purity and state of the tissue at the time of processing, and the targeted ECM final product components. Most decellularization processes disrupt ECM integrity to a certain extent to remove nuclear content and the processing of the dECM to create reconstituted hydrogels can further impact ECM integrity and activity (176, 183). Altogether, combinatory ECM protein hydrogels promote a dynamic reciprocity that can be highly beneficial for understanding cell-cell and cell-matrix interactions within an islet organoid and supporting the stable long-term culture of islets.

### 4.2 Microphysiological Systems

The static culture of multi-cellular islet organoids is challenged by insufficient nutritional gradients created by their high metabolic activity and reliance on diffusion-based transport (184). Transport efficiency is further dampened when islets are placed within 3D hydrogels. Culture systems such as stirred flasks, rotating wall vessels, hollow fibers, and direct perifusion cambers, can support 3D culture by promoting convective-based mass transport for a more effective exchange of nutrients and waste. While likely desirable for the scale-up of cell cultures for translational use, these techniques require larger culture media volumes when compared to typical 2D culture. Further, they exhibit limited capacities for discreet built-in assays for studying islet pathophysiology, pharmaceutical agent screenings, or conducting mechanism of action studies. Microphysiological systems (MPS), also known as tissue chips or organs-on-chips, impart discreet convective flow at a small culture scale, while also facilitating the integration of sensors, outputs, and optical monitoring within a highly defined and controlled microenvironment. MPS mimicking key aspects of organs such as the lung, heart, kidney, and vasculature are becoming widely available within the scientific community (185, 186).

The *in vitro* recreation of an islet-niche demands the precise control of various culture conditions, ranging from temperature, pH, humidity, and oxygenation. In addition, a predictable fluidic flow ensures efficient nutrient delivery, as well as provides biomechanical cues and forces onto the cells within the larger islet cluster [**Figure 3B** (i)]. Beyond delivering more physiological and reproducible culture conditions, this deliberate control provides expansive capacities to precisely perturb and interrogate the islet within the MPS. Various cell culture conditions and geometries are feasible within the islet MPS, which facilitate the incorporation of diverse cell types and morphology (*i.e.*, homogenous versus heterogeneous cell population, monolayers versus 3D clusters), 3D hydrogels capable of supporting both islet clusters and/or vascular structures, and fluidic shear stress (*i.e.*, gravity-based flow, peristaltic- or pressure-based flow) [**Figure 3B** (ii)]. For instance, 2D homogeneous or heterogeneous cell monolayers can be cultured within an MPS to interrogate cell-cell and cell-matrix observations at a subcellular resolution with the added complexity of cell shear stress. Furthermore, monolayers can be cultured onto standard plastic or ECM-coated surfaces to investigate the impact of 2D cell-matrix interactions. Alternatively, in accord with their native 3D structure, islet 3D clusters can be freely cultured within the MPS niche. Culturing islets alone within the MPS can provide convective delivery of nutrients and removal of waste, as well as permit temporal tracking of cellular dynamics. The inclusion of ECM cues and 3D support to islets can further bio-augment the system to support the dynamic complex structures required for cell adhesion, growth, and responsiveness. Islet-matrix interactions can be integrated by either coating the islet surface with relevant ECM (187), submerging islets within a thin layer of hydrogel (188), or fully encasing the islets within a hydrogel (41) [**Figure 3B** (ii)]. Conceivably, when culturing hydrogel-embedded islets, the presence of flow plays a crucial part in an effective nutrient exchange, as deleterious diffusional gradients can be created by a macroscale hydrogel (156). To provide modularity in the selection of geometric, cellular, and matrix features to support broad hypothesis testing, MPS design features should be highly adaptable.

Tailoring the culturing capacity of islets within an MPS is not a frivolous feat. Isolated islets cultured in adhesive cell culture platforms lose their 3D morphology and function due to cell spreading. Alternatively, if they are incubated on a geometrically straight/flat non-adhesive space, then they could wash right out with the fluidic flow. Thus, an islet MPS must contain a design where the islets can be entrapped and immobilized within a niche (**Figure 3C**). The entrapment of islets can be achieved by using device geometry or/and 3D hydrogel encapsulation. Single-well geometries (189–195) or the manipulation of channel heights (196) can be used to physically entrap single and encapsulated islets as they are loaded into the system, which permits the intimate interrogation of single islets [**Figure 3C** (i)]. This approach can be scaled up to create multiple traps, that are geometrically aligned [**Figure 3C** (ii)] (197–204). While this can increase the culture capacity of the system, the total number of islets that can be cultured is limited to the order of tens of islets. Alternatively, a millimeter-scale – open-well system approach can assess larger scales (*i.e.*, 10 to 100 islets) [**Figure 3C** (iii)] (156, 188, 198, 205–209). In this approach, more aggregated metrics are collected, although single islet imaging is still feasible. Islet retrievability is also feasible in this open well system, where samples can be used for post-culture analysis. A challenge with this approach is mitigating the clumping of clusters over time, but this can be resolved with the use of 3D hydrogels (156) or an open well system with small geometric well-traps that capture single clusters [**Figure 3C** (iv)] (210–212). Overall, the architectural features within the islet MPS is a critical parameter in its and is riven by the key metrics of interest (*e.g.*, single cluster or aggregate-based), as well as the driving hypothesis of the work.

The materials used for manufacturing an islet MPS is a key parameter of interest because the surface chemistry of the device can influence the cell biology, hydrodynamics, and the transport and absorption of compounds. One of the most commonly used materials for fabricating MPS is polydimethylsiloxane (PDMS), due to its ease in fabricating custom molds, optical transparency, and gas permeability (185, 213, 214). For islet culture, several PDMS-based MPS have been reported (188, 196, 199, 206, 210, 212). Despite its widespread use, PDMS material properties, such as adsorption of small molecules and the leaching of uncrosslinked oligomers, restrict its utility for scientific discovery applications (215–217). The photolithographic fabrication of PDMS-based MPS can also impart inherent size restrictions. While this does not create a problem for 2D cell culture techniques, it does restrict the utilization of MPS for 3D organoids with sizes ranging from 50 µm to 1 mm (111, 188). Glass is an alternative material used to fabricate MPS, providing the advantages of supporting surface chemistry pretreatments, glass etching, and electroosmotic flow (190, 191, 193, 218); however, the use of this material, particularly for rapid prototyping and scalable MPS manufacturing, can be arduous. Alternatively, thermoplastics, such as polystyrene, poly(methyl-methacrylate), and polycarbonate, support scalable manufacturing and avoid the challenges of biofouling (219). Islet-specific MPS made from thermoplastics have demonstrated preservation of islet function while supporting *in situ* imaging and measurements of secreted hormones (156, 200, 205). With that in mind, the material selected for the fabrication of an islet MPS should serve to permit rapid prototyping, large-scale manufacturing, and durable use without sacrificing its intended use for culturing islet organoids.

Another key MPS advantage is the integration of built-in assays that conveniently report the current health status of the residing cells. *In situ* and off-line measurements of cells is essential in the study of endocrine tissues, as they rely on accurate measurements of secreted hormones and cellular readouts. Thus, the value of a next-generation islet MPS can be greatly extended by incorporating *in situ* spatio-temporal interrogation of micro-assays to monitor cells within the engineered niche (**Figure 3B**). This can be accomplished by designing MPS prototypes that support high-resolution imaging modalities. Such imaging capacities can track cellular destruction, functional readings, as well as interactions with the surrounding matrix or other cell types, in real-time while under continuous perifusion. In engineering an islet MPS, it is recommended that desired readouts are first defined, as the MPS architectural design and the material of choice can sometimes dictate the feasibility of assay integration. For example, an islet MPS made from thermoplastics with a desire to measure fluorescently-labeled insulin required the modification of the islet niche to include borosilicate glass (200). An essential assay for most platforms is the capacity to monitor the dynamic responses of islets to physiologic stimuli, such as a glucose challenge. To date, methods of on-demand monitoring of islet function can be achieved through capillary electrophoresis immunoassays (220–222) or intracellular calcium oscillation monitoring as a surrogate for insulin secretion (138, 223). Recent attraction in leveraging bioreporters to design cellular biosensors is another great avenue to dynamically monitor the biological activities of islets during long-term culture. In general, cells can be virally transfected or genetically altered to induce modifications in proteins that “bioreport” specific activities. These biosensor cells can be added to the culture platform or the islets themselves can be modified to serve as biosensors. For instance, islets can be transfected with GCaMP6 adenovirus for *in situ* reporting of intra-cellular calcium activities (113, 156). Alternatively, distinct biosensors (*i.e.*, cell lines) that report specific soluble metabolites can be incorporated into the downstream flowstream. Islet-specific offline biosensors can monitor hormones and neurotransmitters such as serotonin (224), GABA (γ-aminobutyric acid) (225), glutamate (226), acetylcholine (227), and ROS (228). Additional resources for cell-based biosensors and electrochemical biosensors are nicely reviewed elsewhere (229, 230). The desire to include built-in assays and biosensors must be considered cautiously to not abolish the functionality, durability, and robustness of the islet MPS. Often, they require a separate set of reagents and ploys that may not be easily incorporated within the niche being built for the islet organoids. In that case, approaches that support independent or exchangeable modules may avoid the conflict. Islet MPS built to include such modular assay kits can be of high value to the islet biologists and immunologists to interrogate key biological questions as it pertains to islet biology and pathophysiology.

## 5 The Progenies of 3D Islet MPS

At the time of writing, there is no “has-it-all” approach when engineering a diabetes disease model. Thus, it becomes essential for engineers to define the aspect(s) of the native islet niche that are being modeled or recreated. This review, in conjunction with other key review papers recently reported (65, 231–233), should provide guidance for identifying important aspects of islet physiology to include in a given engineered islet niche. Altogether, the augmentation of the next generation of islet MPS demands inclusion of the following criteria: i) purposeful geometric designs, ii) 3D hydrogels, iii) controlled perifusion, and iv) robust workflow and interrogation capacities customized to the specific needs for the islet.

Specifically, islet MPS designs cannot encompass flat geometries with straight paths and channels that are common for 2D cultures, as 2D monolayers of dissociated islets exhibit poor physiological phenotype and function when compared to their 3D counterparts. Culturing of 3D islets demands sophisticated designs that immobilize the clusters from washing out while they are subjected to continuous flow. The height of the islet MPS device must support the culture of organoids in the range of 100-500 µm in diameter. The surface typography of the device must also be non-adherent over extended culture periods; otherwise, islets may adhere and lose their native 3D morphology. Long-term cultures may require additional attention, as even non-adherent surfaces can foul over time and become adherent.

Next, the incorporation of certain aspects of native 3D architectures by using hydrogels is important for durable *in vitro* culture. 3D hydrogels can provide attachment sites for cells and can serve as a platform for the inclusion or development of additional 3D structures, such as vasculature networks [**Figure 3B** (ii)]. Hydrogels can also enhance 3D imaging, as they stably immobilize the 3D cell cluster. Hydrogel selection is likely driven by the specific hypothesis or goals of the MPS. For example, a hydrogel supportive of vascular development is distinctly different from one seeking to support durable 3D islet encapsulation. The need to study additional dynamic features, for example, the tracking islet-immune interactions, would require a 3D hydrogel that is supportive to both islet suspension and T cell motility. Finally, while there is an increased interest in developing multi-component hydrogels that resemble the heterogenic nature of the native islet-BM niche, the development of the next generation hydrogels is limited by our knowledge of identifying true proportions and compositions of the islet-specific ECM protein niche. By filling this knowledge gap, we would be able to engineer superior hydrogels for enhanced islet culture.

The islet MPS must allow for the controlled perifusion of islets with fully developed laminar flow. Islets are highly metabolically active clusters that require a robust supply of nutrients and oxygen, as well as an effective means to clear metabolic waste. This is especially critical for impure batches of islets or for living pancreatic slices, where acinar tissue can accumulate enzymes proximal to the islet niche. A carefully crafted islet MPS niche should present continuous convective forces to support this goal. The fluidic stream should contact the islet surface rather than flow from a distance away. Such exposure can present mechano-transduction forces on the surface of single cells within an islet. Inducing shear stresses on the islet surface can initiate a cascade of focal adhesion kinases, which could reduce activation of apoptosis, and ultimately support islet function (234). The scale of shear stresses induced in the MPS must be carefully tailored, however, as there are risks of shear-induced β-cell damage (212, 235). The customized perifusion of islets can give additional insight into the dynamics of key hormones and metabolites with temporal readout resolutions. One can stimulate islet organoids with physio-mimetic challenges and evaluate their performance. It can also allow for the exploration of gradient impact versus local toxicity effects of deleterious agents (*e.g.*, cytokines, ROS) near the islets. Overall, the controlled perifusion of islets can provide discreet and controlled flow with the presence of heterogenic features of the peripheral islet microenvironment and can serve as a gold standard tool to perform basic scientific research.

The progenies of 3D islet MPS must have robust handling and *in situ* imaging capacity. The islet MPS should allow the adaptable and customizable workflow to the users. Considerations must be made to ease the islet loading processes and, concurrently, retrievability should be enabled for access to genomic-, flow-cytometry based-, and/or super-resolution imaging- data. Integration of *in situ* imaging capacities must be furnished to enable insightful spatiotemporal tracking and/or optical stimulation capacity while simultaneously under perfusion and within an environmentally controlled chamber. The MPS should also allow ease in introducing a series of media, drugs, and/or cells to imitate hypothesized biological events. The fabrication and use of islet MPS should also be reasonable for its scalable production sustainably and economically without sacrificing its utility. Altogether, a carefully orchestrated islet MPS design should deliver a “work-horse” platform for testing a broad spectrum of hypotheses.

The 3D islet MPS can be further equipped with added features and functionality by including multiple cell types, automation processes, and expansive assay capacities. Features that permit the incorporation of different solutes, media, and multiple cell types can elevate the impact of the islet MPS (**Figure 3B (ii)**). Continuous recirculation of different solutes (*i.e.*, drug molecules) or even cells (*i.e.*, immune cells) may enable dynamic interrogation of islet pharmacokinetics and disease pathology. For example, in the case of understanding islet-immune attack during diabetes pathogenesis, such advanced models can highlight and impart phenomenon that may be completely different when studied under static culture conditions or even under a gravity-based flow. Another attractive component of creating a relevant 3D islet niche is the incorporation of a fully vascularized cavity surrounding the cultured islets [**Figure 3B** (ii)]. A vascularized 3D islet MPS could support the investigation into the role of ECs and islet connections on islet physiology *in vitro*. Anastomosis of engineered vascular network with the resident intra-islet capillaries could further lead to biorelevant islet MPS models. Such models could be used to investigate the role of capillary flow in β-cell stimulation/secretion as well as for our understanding of how immune cells transverse through micro-vessels and migrate towards targeted islet cells. An additional transformative resource is the utilization of isogenic cells within an advanced 3D islet MPS. Using hiPSCs, the feasibility of creating a multi-cellular repertoire from the same human source opens new avenues for creating patient-specific disease models containing not only pancreatic islet cells, but immune cell components (*e.g.*, macrophages, T cells, and B cells) (122). In addition, automation strategies for inputs and outputs for cells, hydrogels, and media can streamline MPS utility by increasing robustness and user adaptability. Lastly, expansive assay capacities either encompassing “all-in-one” islet MPS or completely modular interrogation schemes can boost the impact of a 3D islet MPS. For example, *in situ* oxygen-sensing strategies combined with hormonal or stress-signal measurements *via* biosensors can have broader applications in islet research for not only the temporal *in situ* monitoring of islet health but also for understanding factors that regulate their fate. Modularity in biosensor platforms can also be designed such that they can be assembled for an on-demand interrogation.

## 6 Conclusion

Overall, the next era of islet research is being driven by ambitious endeavors supported by engineers, biologists, and immunologists. By bridging the gap between different stakeholders to ultimately progress diabetes research, it is the hope that this roadmap provides a guide for engineers to craft progenies of an islet MPS that are bio-relevant and easily adaptable by the broader scientific community. Concurrently, we hope that biologists and immunologists are inspired to use engineering principles and tools to ask sophisticated questions. Since the revelation of insulin nearly a century ago, undoubtedly, there have been prominent efforts made in understanding diabetes. As the next century unfolds itself with the revolution in life sciences and biotechnology, we posit that a focus on engineering sophisticated benchtop platforms for studying islets will accelerate the timeline for realizing a cure for diabetes.

## Author Contributions

SNP conceived the idea for the review paper. SNP and CLS wrote the manuscript with critical input from CEM. SNP designed the overall visualization of the figures with feedback from CLS. RC illustrated the native islet figure with feedback from SNP and CLS. All authors read and approved the final version of the manuscript.

## Funding

This work was supported by NIDDK-supported Human Islet Research Network (HIRN; https://hirnetwork.org/; UG3DK122638) and NIH grants RO1 DK126413 and RO1 DK127497.

## Conflict of Interest

The authors declare that the research was conducted in the absence of any commercial or financial relationships that could be construed as a potential conflict of interest.

## Publisher’s Note

All claims expressed in this article are solely those of the authors and do not necessarily represent those of their affiliated organizations, or those of the publisher, the editors and the reviewers. Any product that may be evaluated in this article, or claim that may be made by its manufacturer, is not guaranteed or endorsed by the publisher.

## Abbreviations

T1D, Type 1 diabetes; T2D, Type 2 diabetes; ECM, Extracellular matrix; BM, Basement membrane ECM; IM, Interstitial membrane ECM; ECs, Endothelial cells; ROS, Reactive oxygen species; hiPSC, human induced pluripotent stem cells; SC-βCs, Stem cell-derived β-like-cells; 3D, three-dimensional; MPS, microphysiological systems.

## References

[1] Worldwide toll of diabetes. (2021). Available at: https://www.diabetesatlas.org/en/sections/worldwide-toll-of-diabetes.html (Accessed 16th June 2021).

[2] Concannon P Onengut-Gumuscu S Todd JA Smyth DJ Pociot F Bergholdt R. A Human Type 1 Diabetes Susceptibility Locus Maps to Chromosome 21q22.3. Diabetes (2008) 57:2858. doi: 10.2337/db08-0753 18647951 PMC2551699

[3] Zhu M Xu K Chen Y Gu Y Zhang M Luo F. Identification of Novel T1D Risk Loci and Their Association With Age and Islet Function at Diagnosis in Autoantibody-Positive T1D Individuals: Based on a Two-Stage Genome-Wide Association Study. Diabetes Care (2019) 42:1414–21. doi: 10.2337/dc18-2023 31152121

[4] Roep BO Thomaidou S van Tienhoven R Zaldumbide A. Type 1 Diabetes Mellitus as a Disease of the β-Cell (do Not Blame the Immune System?). Nat Rev Endocrinol (2020) 17:150–61. doi: 10.1038/s41574-020-00443-4 PMC7722981 33293704

[5] Eizirik DL Pasquali L Cnop M. Pancreatic β-Cells in Type 1 and Type 2 Diabetes Mellitus: Different Pathways to Failure. Nat Rev Endocrinol (2020) 16:349–62. doi: 10.1038/s41574-020-0355-7 32398822

[6] Kim A Miller K Jo J Kilimnik G Wojcik P Hara M. Islet Architecture: A Comparative Study. Islets (2009) 1(2):129–36. doi: 10.4161/isl.1.2.9480 PMC2894473 20606719

[7] Dolenšek J Rupnik MS Stožer A. Structural Similarities and Differences Between the Human and the Mouse Pancreas. Islets (2015) 7(1):e1024405. doi: 10.1080/19382014.2015.1024405 26030186 PMC4589993

[8] Paniccia A Schulick RD. Pancreatic Physiology and Functional Assessment. In: Blumgart’s Surg. Liver, Biliary Tract Pancreas Sixth Ed Elsevier, vol. 1–2. (2017). p. 66–76.e3. doi: 10.1016/B978-0-323-34062-5.00004-2

[9] Ionescu-Tirgoviste C Gagniuc PA Gubceac E Mardare L Popescu I Dima S. A 3D Map of the Islet Routes Throughout the Healthy Human Pancreas. Sci Rep (2015) 5:1–14. doi: 10.1038/srep14634 PMC4586491 26417671

[10] Saito K Iwama N Takahashi T. Morphometrical Analysis on Topographical Difference in Size Distribution, Number and Volume of Islets in the Human Pancreas. Tohoku J Exp Med (1978) 124:177–86. doi: 10.1620/tjem.124.177 347635

[11] Pisania A Weir GC O'Neil JJ Omer A Tchipashvili V Lei J. Quantitative Analysis of Cell Composition and Purity of Human Pancreatic Islet Preparations. Lab Investig (2010) 90:1661–75. doi: 10.1038/labinvest.2010.124 PMC2966538 20697378

[12] Buchwald P Wang X Khan A Bernal A Fraker C Inverardi L. Quantitative Assessment of Islet Cell Products: Estimating the Accuracy of the Existing Protocol and Accounting for Islet Size Distribution. Cell Transpl (2009) 18:1223–35. doi: 10.3727/096368909X476968 19818209

[13] Steiner DJ Kim A Miller K Hara M. Pancreatic Islet Plasticity: Interspecies Comparison of Islet Architecture and Composition. Islets (2010) 2:135. doi: 10.4161/isl.2.3.11815 20657742 PMC2908252

[14] Dybala MP Hara M. Heterogeneity of the Human Pancreatic Islet. Diabetes (2019) 68:1230–9. doi: 10.2337/db19-0072 PMC6610021 30936150

[15] Noguchi GM Huising MO. Integrating the Inputs That Shape Pancreatic Islet Hormone Release. Nat Metab (2019) 1:1189–201. doi: 10.1038/s42255-019-0148-2 PMC7378277 32694675

[16] Townsend SE Gannon M. Extracellular Matrix–Associated Factors Play Critical Roles in Regulating Pancreatic β-Cell Proliferation and Survival. Endocrinology (2019) 160:1885–94. doi: 10.1210/en.2019-00206 PMC6656423 31271410

[17] Jansson L Barbu A Bodin B Drott CJ Espes D Gao X. Pancreatic Islet Blood Flow and its Measurement. Ups J Med Sci (2016) 121:81–95. doi: 10.3109/03009734.2016.1164769 27124642 PMC4900068

[18] In’t Veld P Marichal M. Microscopic Anatomy of the Human Islet of Langerhans. Adv Exp Med Biol (2010) 654:1–19. doi: 10.1007/978-90-481-3271-3_1 20217491

[19] Brereton MF Vergari E Zhang Q Clark A. Alpha-, Delta- and PP-Cells: Are They the Architectural Cornerstones of Islet Structure and Co-Ordination? J Histochem Cytochem (2015) 63(8):575–91. doi: 10.1369/0022155415583535 PMC4530398 26216135

[20] Irving-Rodgers HF Ziolkowski AF Parish CR Sado Y Ninomiya Y Simeonovic CJ. Molecular Composition of the Peri-Islet Basement Membrane in NOD Mice: A Barrier Against Destructive Insulitis. Diabetologia (2008) 51:1680–8. doi: 10.1007/s00125-008-1085-x PMC2516190 18633594

[21] Korpos É Kadri N Kappelhoff R Wegner J Overall CM Weber E. The Peri-Islet Basement Membrane, a Barrier to Infiltrating Leukocytes in Type 1 Diabetes in Mouse and Human. Diabetes (2013) 62:531–42. doi: 10.2337/db12-0432 PMC3554379 23139348

[22] Kaido T Yebra M Cirulli V Montgomery AM. Regulation of Human β-Cell Adhesion, Motility, and Insulin Secretion by Collagen IV and its Receptor α1β1. J Biol Chem (2004) 279:53762–9. doi: 10.1074/jbc.M411202200 15485856

[23] Virtanen I Banerjee M Palgi J Korsgren O Lukinius A Thornell L-E. Blood Vessels of Human Islets of Langerhans are Surrounded by a Double Basement Membrane. Diabetologia (2008) 51:1181–91. doi: 10.1007/s00125-008-0997-9 18438639

[24] Bogdani M Korpos E Simeonovic CJ Parish CR Sorokin L Wight TN. Extracellular Matrix Components in the Pathogenesis of Type 1 Diabetes. Curr Diabetes Rep (2014) 14:1–11. doi: 10.1007/s11892-014-0552-7 PMC4238291 25344787

[25] Nikolova G Jabs N Konstantinova I Domogatskaya A Tryggvason K Sorokin L. The Vascular Basement Membrane: A Niche for Insulin Gene Expression and β Cell Proliferation. Dev Cell (2006) 10:397–405. doi: 10.1016/j.devcel.2006.01.015 16516842

[26] Otonkoski T Banerjee M Korsgren O Thornell L-E Virtanen I. Unique Basement Membrane Structure of Human Pancreatic Islets: Implications for β-Cell Growth and Differentiation. Diabetes Obes Metab (2008) 10:119–27. doi: 10.1111/j.1463-1326.2008.00955.x 18834439

[27] Geutskens SB Homo-Delarche F Pleau J-M Durant S Drexhage HA Savino W. Extracellular Matrix Distribution and Islet Morphology in the Early Postnatal Pancreas: Anomalies in the non-Obese Diabetic Mouse. Cell Tissue Res (2004) 318:579–89. doi: 10.1007/s00441-004-0989-0 15480796

[28] Stendahl JC Kaufman DB Stupp SI. Extracellular Matrix in Pancreatic Islets: Relevance to Scaffold Design and Transplantation. Cell Transplant (2009) 18:1–12. doi: 10.3727/096368909788237195 19476204 PMC2724969

[29] Smink AM de Vos P. Therapeutic Strategies for Modulating the Extracellular Matrix to Improve Pancreatic Islet Function and Survival After Transplantation. Curr Diabetes Rep (2018) 18:1–7. doi: 10.1007/s11892-018-1014-4 PMC5960477 29779190

[30] Takahashi Y Sekine K Kin T Takebe T Taniguchi H. Self-Condensation Culture Enables Vascularization of Tissue Fragments for Efficient Therapeutic Transplantation. Cell Rep (2018) 23:1620–9. doi: 10.1016/j.celrep.2018.03.123 PMC8289710 29742420

[31] Figliolini F Cantaluppi V De Lena M Beltramo S Romagnoli R Salizzoni M. Isolation, Characterization and Potential Role in Beta Cell-Endothelium Cross-Talk of Extracellular Vesicles Released From Human Pancreatic Islets. PLoS One (2014) 9:e102521. doi: 10.1371/journal.pone.0102521 25028931 PMC4100900

[32] Kilkenny DM Rocheleau JV. Fibroblast Growth Factor Receptor-1 Signaling in Pancreatic Islet β-Cells Is Modulated by the Extracellular Matrix. Mol Endocrinol (2008) 22:196–205. doi: 10.1210/me.2007-0241 17916654 PMC2194636

[33] Nilsson J Fardoos R Hansen L Lövkvist H Pietras K Holmberg D. Recruited Fibroblasts Reconstitute the Peri-Islet Membrane: A Longitudinal Imaging Study of Human Islet Grafting and Revascularisation. Diabetologia (2020) 63:137–48. doi: 10.1007/s00125-019-05018-1 PMC6890581 31701200

[34] Beattie GM Montgomery AMP Lopez AD Hao E Perez B Just ML. A Novel Approach to Increase Human Islet Cell Mass While Preserving β-Cell Function. Diabetes (2002) 51:3435–9. doi: 10.2337/diabetes.51.12.3435 12453897

[35] Navarro-Alvarez N Rivas-Carrillo JD Soto-Gutierrez A Yuasa T Okitsu T Noguchi H. Reestablishment of Microenvironment is Necessary to Maintain *In Vitro* and *In Vivo* Human Islet Function. Cell Transpl (2008) 17:111–9. doi: 10.3727/000000008783907125 18468241

[36] Pinkse GGM Bouwman WP Jiawan-Lalai R Terpstra OT Bruijn JA deHeer E. Integrin Signaling *via* RGD Peptides and Anti-β1 Antibodies Confers Resistance to Apoptosis in Islets of Langerhans. Diabetes (2006) 55:312–7. doi: 10.2337/diabetes.55.02.06.db04-0195 16443762

[37] Wang RN Rosenberg L. Maintenance of Beta-Cell Function and Survival Following Islet Isolation Requires Re-Establishment of the Islet-Matrix Relationship. J Endocrinol (1999) 163:181–90. doi: 10.1677/joe.0.1630181 10556766

[38] Zhu Y Chen S Liu W Zhang L Xu F Hayashi T. Collagens I and V Differently Regulate the Proliferation and Adhesion of Rat Islet INS-1 Cells Through the Integrin β1/E-Cadherin/β-Catenin Pathway. Connect Tissue Res (2021) 62(6):658–70. doi: 10.1080/03008207.2020.1845321 33957832

[39] Maillard E Juszczak MT Clark A Hughes SJ Gray DRW Johnson PRV. Perfluorodecalin-Enriched Fibrin Matrix for Human Islet Culture. Biomaterials (2011) 32:9282–9. doi: 10.1016/j.biomaterials.2011.08.044 21899883

[40] Yap WT Salvay DM Silliman MA Zhang X Bannon ZG Kaufman DB. Collagen IV-Modified Scaffolds Improve Islet Survival and Function and Reduce Time to Euglycemia. Tissue Eng - Part A (2013) 19:2361–72. doi: 10.1089/ten.tea.2013.0033 PMC3807710 23713524

[41] Jiang K Chaimov D Patel SN Liang J-P Wiggins SC Samojlik MM. 3-D Physiomimetic Extracellular Matrix Hydrogels Provide a Supportive Microenvironment for Rodent and Human Islet Culture. Biomaterials (2019) 198:37–48. doi: 10.1016/j.biomaterials.2018.08.057 30224090 PMC6397100

[42] Cross SE Vaughan RH Willcox AJ McBride AJ Abraham AA Han B. Key Matrix Proteins Within the Pancreatic Islet Basement Membrane Are Differentially Digested During Human Islet Isolation. Am J Transpl (2017) 17:451–61. doi: 10.1111/ajt.13975 27456745

[43] Bogdani M Johnson PY Potter-Perigo S Nagy N Day AJ Bollyky PL. Hyaluronan and Hyaluronan-Binding Proteins Accumulate in Both Human Type 1 Diabetic Islets and Lymphoid Tissues and Associate With Inflammatory Cells in Insulitis. Diabetes (2014) 63:2727–43. doi: 10.2337/db13-1658 PMC4113060 24677718

[44] Lifson N Kramlinger KG Mayrand RR Lender EJ. Blood Flow to the Rabbit Pancreas With Special Reference to the Islets of Langerhans. Gastroenterology (1980) 79:466–73. doi: 10.1016/0016-5085(80)90371-6 7000613

[45] Fraser PA Henderson JR. The Arrangement of Endocrine and Exocrine Pancreatic Microcirculation Observed in the Living Rabbit. Q J Exp Physiol Cogn Med Sci (1980) 65:151–8. doi: 10.1113/expphysiol.1980.sp002499 6997917

[46] Lifson N Lassa CV. Note on the Blood Supply of the Ducts of the Rabbit Pancreas. Microvasc Res (1981) 22:171–6. doi: 10.1016/0026-2862(81)90086-8 7033729

[47] Jansson L Hellerström C. Stimulation by Glucose of the Blood Flow to the Pancreatic Islets of the Rat. Diabetologia (1983) 25:45–50. doi: 10.1007/BF00251896 6350083

[48] Carlsson PO Olsson R Källskog Ö Bodin B Andersson A Jansson L. Glucose-Induced Islet Blood Flow Increase in Rats: Interaction Between Nervous and Metabolic Mediators. Am J Physiol - Endocrinol Metab (2002) 283:E457–64. doi: 10.1152/ajpendo.00044.2002 12169438

[49] Dybala MP Gebien LR Reyna ME Yu Y Hara M. Implications of Integrated Pancreatic Microcirculation: Crosstalk Between Endocrine and Exocrine Compartments. Diabetes (2020) 69:2566–74. doi: 10.2337/db20-0810 PMC7679783 33148810

[50] Dybala MP Kuznetsov A Motobu M Hendren-Santiago BK Philipson LH Chervonsky AV. Integrated Pancreatic Blood Flow: Bidirectional Microcirculation Between Endocrine and Exocrine Pancreas. Diabetes (2020) 69:1439–50. doi: 10.2337/db19-1034 PMC7306124 32198213

[51] Dionne KE Colton CK Lyarmush M. Effect of Hypoxia on Insulin Secretion by Isolated Rat and Canine Islets of Langerhans. Diabetes (1993) 42:12–21. doi: 10.2337/diab.42.1.12 8420809

[52] Papas KK Bellin MD Sutherland DER Suszynski TM Kitzmann JP Avgoustiniatos ES. Islet Oxygen Consumption Rate (OCR) Dose Predicts Insulin Independence in Clinical Islet Autotransplantation. PLoS One (2015) 10:e0134428. doi: 10.1371/journal.pone.0134428 26258815 PMC4530873

[53] MA S ME F. Interstitial Flow and its Effects in Soft Tissues. Annu Rev Biomed Eng (2007) 9:229–56. doi: 10.1146/annurev.bioeng.9.060906.151850 17459001

[54] Korsgren E Korsgren O. An Apparent Deficiency of Lymphatic Capillaries in the Islets of Langerhans in the Human Pancreas. Diabetes (2016) 65:1004–8. doi: 10.2337/db15-1285 26822093

[55] O’morchoe CCC. Lymphatic System of the Pancreas. Microsc Res Tech (1997) 37:456–77. doi: 10.1002/(SICI)1097-0029(19970601)37:5/6<456::AID-JEMT9>3.0.CO;2-B 9220424

[56] Murakami T Fujita T Taguchi T Nonaka Y Orita K. The Blood Vascular Bed of the Human Pancreas, With Special Reference to the Insulo-Acinar Portal System. Scanning Electron Microscopy of Corrosion Casts. Arch Histol Cytol (1992) 55:381–95. doi: 10.1679/aohc.55.381 1482603

[57] Almaça J Weitz J Rodriguez-Diaz R Pereira E Caicedo A. The Pericyte of the Pancreatic Islet Regulates Capillary Diameter and Local Blood Flow. Cell Metab (2018) 27:630–44.e4. doi: 10.1016/j.cmet.2018.02.016 29514070 PMC5876933

[58] Henderson JR Moss MC. A Morphometric Study of the Endocrine and Exocrine Capillaries of the Pancreas. Q J Exp Physiol (1985) 70:347–56. doi: 10.1113/expphysiol.1985.sp002920 3898188

[59] Bonner-Weir S. Morphological Evidence for Pancreatic Polarity of β-Cell Within Islets of Langerhans. Diabetes (1988) 37:616–21. doi: 10.2337/diabetes.37.5.616 3282948

[60] Cohrs CM Chen C Jahn SR Stertmann J Chmelova H Weitz J. Vessel Network Architecture of Adult Human Islets Promotes Distinct Cell-Cell Interactions *In Situ* and Is Altered After Transplantation. Endocrinology (2017) 158:1373–85. doi: 10.1210/en.2016-1184 28324008

[61] Bosco D Armanet M Morel P Niclauss N Sgroi A Muller YD. Unique Arrangement of α- and β-Cells in Human Islets of Langerhans. Diabetes (2010) 59:1202–10. doi: 10.2337/db09-1177 PMC2857900 20185817

[62] Geron E Boura-Halfon S Schejter ED Shilo BZ. The Edges of Pancreatic Islet β Cells Constitute Adhesive and Signaling Microdomains. Cell Rep (2015) 10:317–25. doi: 10.1016/j.celrep.2014.12.031 25600867

[63] Gan WJ Zavortink M Ludick C Templin R Webb R Webb R. Cell Polarity Defines Three Distinct Domains in Pancreatic β-Cells. J Cell Sci (2017) 130:143–51. doi: 10.1242/jcs.185116 PMC5394774 26919978

[64] Gan WJ Do OH Cottle L Ma W Kosobrodova E Cooper-Whitel J. Local Integrin Activation in Pancreatic β Cells Targets Insulin Secretion to the Vasculature. Cell Rep (2018) 24:2819–26.e3. doi: 10.1016/j.celrep.2018.08.035 30208309

[65] Lammert E Thorn P. The Role of the Islet Niche on Beta Cell Structure and Function. J Mol Biol (2020) 432:1407–18. doi: 10.1016/j.jmb.2019.10.032 31711959

[66] Low JT Zavortink M Mitchell JM Gan WJ Do OH Schwiening CJ. Insulin Secretion From Beta Cells in Intact Mouse Islets is Targeted Towards the Vasculature. Diabetologia (2014) 57:1655–63. doi: 10.1007/s00125-014-3252-6 PMC4079948 24795086

[67] Singh R Cottle L Loudovaris T Xiao D Yang P Thomas HE. Enhanced Structure and Function of Human Pluripotent Stem Cell-Derived Beta-Cells Cultured on Extracellular Matrix. Stem Cells Transl Med (2020) 10(3):492–505. doi: 10.1002/sctm.20-0224 33145960 PMC7900592

[68] Peiris H Bonder CS Coates PTH Keating DJ Jessup CF. The β-Cell/EC Axis: How do Islet Cells Talk to Each Other? Diabetes (2014) 63:3–11. doi: 10.2337/db13-0617 24357688

[69] Konstantinova I Nikolova G Ohara-Imaizumi M Meda P Kučera T Zarbalis K. EphA-Ephrin-A-Mediated β Cell Communication Regulates Insulin Secretion From Pancreatic Islets. Cell (2007) 129:359–70. doi: 10.1016/j.cell.2007.02.044 17448994

[70] Parnaud G Lavallard V Bedat B Matthey-Doret D Morel P Berney T. Cadherin Engagement Improves Insulin Secretion of Single Human β-Cells. Diabetes (2015) 64:887–96. doi: 10.2337/db14-0257 25277393

[71] Jaques F Jousset H Tomas A Prost A-L Wollheim CB Irminger J-C. Dual Effect of Cell-Cell Contact Disruption on Cytosolic Calcium and Insulin Secretion. Endocrinology (2008) 149:2494–505. doi: 10.1210/en.2007-0974 18218692

[72] Gerdes JM Christou-Savina S Xiong Y Moede T Moruzzi N Karlsson-Edlund P. Ciliary Dysfunction Impairs Beta-Cell Insulin Secretion and Promotes Development of Type 2 Diabetes in Rodents. Nat Commun (2014) 5:1–13. doi: 10.1038/ncomms6308 25374274

[73] Eberhard D Kragl M Lammert E. ‘Giving and Taking’: Endothelial and β-Cells in the Islets of Langerhans. Trends Endocrinol Metab (2010) 21:457–63. doi: 10.1016/j.tem.2010.03.003 20359908

[74] Islet Cell Resource Centers (ICR). (2001). Available at: https://icr.coh.org/default.asp (Accessed 7th April 2021).

[75] National Institute of Diabetes and Digestive and Kidney Diseases (NIDDK). (1986). Available at: https://www.niddk.nih.gov/ (Accessed 7th April 2021).

[76] Integrated Islet Distribution Program (IIDP). (2009). Available at: https://iidp.coh.org/ (Accessed 7th April 2021).

[77] The Human Pancreas Analysis Porgram (HPAP). (2016). Available at: https://hpap.pmacs.upenn.edu/ (Accessed 14th April 2021).

[78] Pancreatlas. (2016). Available at: https://pancreatlas.org/ (Accessed 14th April 2021).

[79] The Human Islet Phenotyping Program (HIPP). (2016). Available at: https://hipp.coh.org/#/dashboard/ (Accessed 14th April 2021).

[80] Diabetes Epigenome Atlas. (2020). Available at: https://www.diabetesepigenome.org/ (Accessed 14th April 2021).

[81] The human proteome in pancreas - The Human Protein Atlas. (2015). Available at: https://www.proteinatlas.org/humanproteome/tissue/pancreas (Accessed 14th April 2021).

[82] JDRF nPOD | Network for Pancreatic Organ Donors with Diabetes. (2007). Available at: https://www.jdrfnpod.org/ (Accessed 14th April 2021).

[83] Prodo Laboratories, Inc. (2006). Available at: https://prodolabs.com/ (Accessed 19th November 2021).

[84] Cohrs CM Panzer JK Drotar DM Enos SJ Kipke N Chen C. Dysfunction of Persisting β Cells Is a Key Feature of Early Type 2 Diabetes Pathogenesis. Cell Rep (2020) 31:107469. doi: 10.1016/j.celrep.2020.03.033 32268101

[85] Krogvold L Edwin B Buanes T Ludvigsson J Korsgren O Hyöty H. Pancreatic Biopsy by Minimal Tail Resection in Live Adult Patients at the Onset of Type 1 Diabetes: Experiences From the DiViD Study. Diabetologia (2014) 57:841–3. doi: 10.1007/s00125-013-3155-y 24429579

[86] Kaddis JS Olack BJ Sowinski J Cravens J Contreras JL Niland JC. Human Pancreatic Islets and Diabetes Research. JAMA - J Am Med Assoc (2009) 301:1580–7. doi: 10.1001/jama.2009.482 PMC3763818 19366778

[87] King AJ. The Use of Animal Models in Diabetes Research. Br J Pharmacol (2012) 166:877. doi: 10.1111/j.1476-5381.2012.01911.x 22352879 PMC3417415

[88] Van Belle TL Taylor P von Herrath MG. Mouse Models for Type 1 Diabetes. Drug Discovery Today Dis Model (2009) 6:41–5. doi: 10.1016/j.ddmod.2009.03.008 PMC2855847 20407588

[89] Chen Y-G Mathews CE Driver JP. The Role of NOD Mice in Type 1 Diabetes Research: Lessons From the Past and Recommendations for the Future. Front Endocrinol (Lausanne) (2018) 0:51. doi: 10.3389/fendo.2018.00051 PMC5829040 29527189

[90] Chatzigeorgiou A Halapas A Kalafatakis K Kamper E. The Use of Animal Models in the Study of Diabetes Mellitus. In Vivo (Brooklyn) (2009) 23:245–58. 19414410

[91] Fang J-Y Lin C-H Huang T-H Chuang S-Y. *In Vivo* Rodent Models of Type 2 Diabetes and Their Usefulness for Evaluating Flavonoid Bioactivity. Nutrients (2019) 11:530. doi: 10.3390/nu11030530 PMC6470730 30823474

[92] Graham ML Schuurman HJ. Validity of Animal Models of Type 1 Diabetes, and Strategies to Enhance Their Utility in Translational Research. Eur J Pharmacol (2015) 759:221–30. doi: 10.1016/j.ejphar.2015.02.054 25814249

[93] Speier S Nyqvist D Cabrera O Yu J Molano RD Pileggi A. Noninvasive *In Vivo* Imaging of Pancreatic Islet Cell Biology. Nat Med (2008) 14:574–8. doi: 10.1038/nm1701 PMC3538807 18327249

[94] Rodriguez-Diaz R Speier S Molano RD Formoso A Gans I Abdulreda MH. Noninvasive *In Vivo* Model Demonstrating the Effects of Autonomic Innervation on Pancreatic Islet Function. Proc Natl Acad Sci USA (2012) 109:21456–61. doi: 10.1073/pnas.1211659110 PMC3535593 23236142

[95] Brusko TM Russ HA Stabler CL. Strategies for Durable β Cell Replacement in Type 1 Diabetes. Science (80- ) (2021) 373:516–22. doi: 10.1126/science.abh1657 PMC8867839 34326233

[96] Luce S Guinoiseau S Gadault A Letourneur F Blondeau B Nitschke P. Humanized Mouse Model to Study Type 1 Diabetes. Diabetes (2018) 67:1816–29. doi: 10.2337/db18-0202 29967002

[97] Cabrera O Berman DM Kenyon NS Ricordi C Berggren P-O Caicedo A. The Unique Cytoarchitecture of Human Pancreatic Islets has Implications for Islet Cell Function. Proc Natl Acad Sci USA (2006) 103:2334–9. doi: 10.1073/pnas.0510790103 PMC1413730 16461897

[98] Brissova M Fowler MJ Nicholson WE Chu A Hirshberg B Harlan DM. Assessment of Human Pancreatic Islet Architecture and Composition by Laser Scanning Confocal Microscopy. J Histochem Cytochem (2005) 53:1087–97. doi: 10.1369/jhc.5C6684.2005 15923354

[99] Tao L Reese TA. Making Mouse Models That Reflect Human Immune Responses. Trends Immunol (2017) 38:181–93. doi: 10.1016/j.it.2016.12.007 28161189

[100] Hohmeier HE Newgard CB. Cell Lines Derived From Pancreatic Islets. Mol Cell Endocrinol (2004) 228:121–8. doi: 10.1016/j.mce.2004.04.017 15541576

[101] Scharfmann R Staels W Albagli O. The Supply Chain of Human Pancreatic β Cell Lines. J Clin Invest (2019) 129:3511–20. doi: 10.1172/JCI129484 PMC6715382 31478912

[102] Lightfoot YL Chen J Mathews CE. Immune-Mediated β-Cell Death in Type 1 Diabetes: Lessons From Human β-Cell Lines. Eur J Clin Invest (2012) 42:1244–51. doi: 10.1111/j.1365-2362.2012.02711.x PMC3703770 22924552

[103] Van Hoof D Mendelsohn AD Seerke R Desai TA German MS. Differentiation of Human Embryonic Stem Cells Into Pancreatic Endoderm in Patterned Size-Controlled Clusters. Stem Cell Res (2011) 6:276–85. doi: 10.1016/j.scr.2011.02.004 21513906

[104] Mendelsohn AD Bernards DA Lowe RD Desai TA. Patterning of Mono- and Multilayered Pancreatic β-Cell Clusters. Langmuir (2010) 26:9943–9. doi: 10.1021/la1004424 PMC2883011 20218546

[105] Gao B Jing C Ng K Pingguan-Murphy B Yang Q. Fabrication of Three-Dimensional Islet Models by the Geometry-Controlled Hanging-Drop Method. Acta Mech Sin Xuebao (2019) 35:329–37. doi: 10.1007/s10409-019-00856-z

[106] Kosobrodova E Gan WJ Kondyurin A Thorn P Bilek MMM. Improved Multiprotein Microcontact Printing on Plasma Immersion Ion Implanted Polystyrene. ACS Appl Mater Interfaces (2018) 10:227–37. doi: 10.1021/acsami.7b15545 29211435

[107] Bensley RR. Studies on the Pancreas of the Guinea Pig. Am J Anat (1911) 12:297–388. doi: 10.1002/aja.1000120304

[108] Moskalewski S. Isolation and Culture of the Islets of Langerhans of the Guinea Pig. Gen Comp Endocrinol (1965) 5:342–53. doi: 10.1016/0016-6480(65)90059-6 14338040

[109] Lacy PE Kostianovsky M. Method for the Isolation of Intact Islets of Langerhans From the Rat Pancreas. Diabetes (1967) 16:35–9. doi: 10.2337/diab.16.1.35 5333500

[110] Scharp DW Kemp CB Knight MJ Ballinger WF Lacy PE. The Use of Ficoll in the Preparation of Viable Islets of Langerhans From the Rat Pancreas. Transplantation (1973) 16:686–9. doi: 10.1097/00007890-197312000-00028 4201956

[111] Ricordi C Lacy PE Finke EH Olack BJ Scharp DW. Automated Method for Isolation of Human Pancreatic Islets. Diabetes (1988) 37:413–20. doi: 10.2337/diab.37.4.413 3288530

[112] Phelps EA Cianciaruso C Santo-Domingo J Pasquier M Galliverti G Piemonti L. Advances in Pancreatic Islet Monolayer Culture on Glass Surfaces Enable Super-Resolution Microscopy and Insights Into Beta Cell Ciliogenesis and Proliferation. Sci Rep (2017) 7:45961. doi: 10.1038/srep45961 28401888 PMC5388888

[113] Walker JT Haliyur R Nelson HA Ishahak M Poffenberger G Aramandla R. Integrated Human Pseudoislet System and Microfluidic Platform Demonstrate Differences in GPCR Signaling in Islet Cells. JCI Insight (2020) 5(10):e137017. doi: 10.1172/jci.insight.137017 PMC7259531 32352931

[114] Papas KK De Leon H Suszynski TM Johnson RC. Oxygenation Strategies for Encapsulated Islet and Beta Cell Transplants. Advanced Drug Delivery Rev (2019) 139:139–56. doi: 10.1016/j.addr.2019.05.002 31077781

[115] Bhagat L Singh VP Hietaranta AJ Agrawal S Steer ML Saluja AK. Heat Shock Protein 70 Prevents Secretagogue-Induced Cell Injury in the Pancreas by Preventing Intracellular Trypsinogen Activation. J Clin Invest (2000) 106:81–9. doi: 10.1172/JCI8706 PMC314357 10880051

[116] Blinman TA Gukovsky I Mouria M Zaninovic V Livingston E Pandol SJ. Activation of Pancreatic Acinar Cells on Isolation From Tissue: Cytokine Upregulation *via* P38 MAP Kinase. Am J Physiol Physiol (2000) 279:C1993–2003. doi: 10.1152/ajpcell.2000.279.6.C1993 11078716

[117] Raposo do Amaral AS do Amaral R Pawlick RL Rodrigues E Costal F Pepper A Galvão FHF. Glutathione Ethyl Ester Supplementation During Pancreatic Islet Isolation Improves Viability and Transplant Outcomes in a Murine Marginal Islet Mass Model. PLoS One (2013) 8:e55288. doi: 10.1371/journal.pone.0055288 23424628 PMC3570543

[118] Negi S Jetha A Aikin R Hasilo C Sladek R Paraskevas S. Analysis of Beta-Cell Gene Expression Reveals Inflammatory Signaling and Evidence of Dedifferentiation Following Human Islet Isolation and Culture. PLoS One (2012) 7:e30415. doi: 10.1371/journal.pone.0030415 22299040 PMC3267725

[119] Paraskevas S Aikin R Maysinger D Lakey JRT Cavanagh TJ Hering B. Activation and Expression of ERK, JNK, and P38 MAP-Kinases in Isolated Islets of Langerhans: Implications for Cultured Islet Survival. FEBS Lett (1999) 455:203–8. doi: 10.1016/S0014-5793(99)00882-0 10437773

[120] Daoud J Petropavlovskaia M Rosenberg L Tabrizian M. The Effect of Extracellular Matrix Components on the Preservation of Human Islet Function *In Vitro* . Biomaterials (2010) 31:1676–82. doi: 10.1016/j.biomaterials.2009.11.057 20015544

[121] Llacua LA Faas MM de Vos P. Extracellular Matrix Molecules and Their Potential Contribution to the Function of Transplanted Pancreatic Islets. Diabetol (2018) 61:1261–72. doi: 10.1007/s00125-017-4524-8 PMC6449002 29306997

[122] Armitage LH Stimpson SE Santostefano KE Sui L Ogundare S Newby BN. Use of Induced Pluripotent Stem Cells to Build Isogenic Systems and Investigate Type 1 Diabetes. Front Endocrinol (Lausanne) (2021) 1376. doi: 10.3389/fendo.2021.737276 PMC8630743 34858326

[123] Pagliuca FW Millman JR Gürtler M Segel M Van Dervort A Ryu JH. Generation of Functional Human Pancreatic β Cells *In Vitro* . Cell (2014) 159:428–39. doi: 10.1016/j.cell.2014.09.040 PMC4617632 25303535

[124] Rezania A Bruin JE Arora P Rubin A Batushansky I Asadi A. Reversal of Diabetes With Insulin-Producing Cells Derived *In Vitro* From Human Pluripotent Stem Cells. Nat Biotechnol (2014) 32:1121–33. doi: 10.1038/nbt.3033 25211370

[125] Russ HA Parent AV Ringler JJ Hennings TG Nair GG Shveygert M. Controlled Induction of Human Pancreatic Progenitors Produces Functional Beta-Like Cells *In Vitro* . EMBO J (2015) 34:1759–72. doi: 10.15252/embj.201591058 PMC4516429 25908839

[126] Hogrebe NJ Augsornworawat P Maxwell KG Velazco-Cruz L Millman JR. Targeting the Cytoskeleton to Direct Pancreatic Differentiation of Human Pluripotent Stem Cells. Nat Biotechnol (2020) 38:460–70. doi: 10.1038/s41587-020-0430-6 PMC7274216 32094658

[127] Mahaddalkar PU Scheibner K Pfluger S Ansarullah Sterr M Beckenbauer J. Generation of Pancreatic β Cells From CD177+ Anterior Definitive Endoderm. Nat Biotechnol (2020) 38:1061–72. doi: 10.1038/s41587-020-0492-5 32341565

[128] Veres A Faust AL Bushnell HL Engquist EN Kenty JH-R Harb G. Charting Cellular Identity During Human *In Vitro* β-Cell Differentiation. Nature (2019) 569:368–73. doi: 10.1038/s41586-019-1168-5 PMC6903417 31068696

[129] Nair GG Liu JS Russ HA Tran S Saxton MS Chen R. Recapitulating Endocrine Cell Clustering in Culture Promotes Maturation of Human Stem-Cell-Derived β Cells. Nat Cell Biol (2019) 21:263–74. doi: 10.1038/s41556-018-0271-4 PMC6746427 30710150

[130] Velazco-Cruz L Song J Maxwell KG Goedegebuure MM Augsornworawat P Hogrebe NJ. Acquisition of Dynamic Function in Human Stem Cell-Derived β Cells. Stem Cell Rep (2019) 12:351–65. doi: 10.1016/j.stemcr.2018.12.012 PMC6372986 30661993

[131] Hogrebe NJ Maxwell KG Augsornworawat P Millman JR. Generation of Insulin-Producing Pancreatic β Cells From Multiple Human Stem Cell Lines. Nat Protoc (2021) 2021:1–35. doi: 10.1038/s41596-021-00560-y PMC8529911 34349281

[132] Millman JR Xie C Van Dervort A Gürtler M Pagliuca FW Melton DA. Generation of Stem Cell-Derived β-Cells From Patients With Type 1 Diabetes. Nat Commun (2016) 7:1–9. doi: 10.1038/ncomms11463 PMC4866045 27163171

[133] Kaestner KH Campbell–Thompson M Dor Y Gill RG Glaser B Kim SK. What is a β Cell? – Chapter I in the Human Islet Research Network (HIRN) Review Series. Mol Metab (2021) 53:101323. doi: 10.1016/J.MOLMET.2021.101323 34416394 PMC8452767

[134] Velazco-Cruz L Goedegebuure MM Millman JR. Advances Toward Engineering Functionally Mature Human Pluripotent Stem Cell-Derived β Cells. Front Bioeng Biotechnol (2020) 8:786. doi: 10.3389/fbioe.2020.00786 32733873 PMC7363766

[135] Tse HM Gardner G Dominguez-Bendala J Fraker CA. The Importance of Proper Oxygenation in 3D Culture. Front Bioeng Biotechnol (2021) 9:241. doi: 10.3389/fbioe.2021.634403 PMC8042214 33859979

[136] Speier S Rupnik M. A Novel Approach to *in Situ* Characterization of Pancreatic β-Cells. Pflugers Arch Eur J Physiol (2003) 446:553–8. doi: 10.1007/s00424-003-1097-9 12774232

[137] Marciniak A Cohrs CM Tsata V Chouinard JA Selck C Stertmann J. Using Pancreas Tissue Slices for *in Situ* Studies of Islet of Langerhans and Acinar Cell Biology. Nat Protoc (2014) 9:2809–22. doi: 10.1038/nprot.2014.195 25393778

[138] Huber MK Drotar DM Hiller H Beery ML Joseph P Kusmartseva I. Observing Islet Function and Islet-Immune Cell Interactions in Live Pancreatic Tissue Slices. J Vis Exp (2021) (170):e62207. doi: 10.3791/62207 PMC8314551 33900291

[139] Huang Y-C Gaisano HY Leung Y-M. Electrophysiological Identification of Mouse Islet α-Cells: From Isolated Single α-Cells to *in Situ* Assessment Within Pancreas Slices. Islets (2011) 3:139–43. doi: 10.4161/isl.3.4.16166 21623173

[140] Panzer JK Hiller H Cohrs CM Almaça J Enos SJ Beery M. Pancreas Tissue Slices From Organ Donors Enable *in Situ* Analysis of Type 1 Diabetes Pathogenesis. JCI Insight (2020) 5(10):e137017. doi: 10.1172/jci.insight.134525 PMC7205437 32324170

[141] Qadir MMF Álvarez-Cubela S Weitz J Panzer JK Klein D Moreno-Hernández Y. Long-Term Culture of Human Pancreatic Slices as a Model to Study Real-Time Islet Regeneration. Nat Commun (2020) 11:1–15. doi: 10.1038/s41467-020-17040-8 32601271 PMC7324563

[142] Stabler CL Li Y Stewart JM Keselowsky BG. Engineering Immunomodulatory Biomaterials for Type 1 Diabetes. Nat Rev Mater (2019) 4:429–50. doi: 10.1038/s41578-019-0112-5 PMC7332200 32617176

[143] Tomei AA Manzoli V Fraker CA Giraldo J Velluto D Najjar M. Device Design and Materials Optimization of Conformal Coating for Islets of Langerhans. Proc Natl Acad Sci (2014) 111:10514–9. doi: 10.1073/pnas.1402216111 PMC4115512 24982192

[144] Phelps EA Templeman KL Thulé PM García AJ. Engineered VEGF-Releasing PEG–MAL Hydrogel for Pancreatic Islet Vascularization. Drug Delivery Trans Res (2015) 5:125–36. doi: 10.1007/s13346-013-0142-2 PMC4366610 25787738

[145] Borg DJ Bonifacio E. The Use of Biomaterials in Islet Transplantation. Curr Diabetes Rep (2011) 11:434–44. doi: 10.1007/s11892-011-0210-2 PMC3167046 21748257

[146] Qi M Gu Y Sakata N Kim D Shirouzu Y Yamamoto C. PVA Hydrogel Sheet Macroencapsulation for the Bioartificial Pancreas. Biomaterials (2004) 25:5885–92. doi: 10.1016/j.biomaterials.2004.01.050 15172501

[147] Lee KY Mooney DJ. Alginate: Properties and Biomedical Applications. Prog Polymer Sci (Oxford) (2012) 37:106–26. doi: 10.1016/j.progpolymsci.2011.06.003 PMC3223967 22125349

[148] Krishnamurthy NV Gimi B. Encapsulated Cell Grafts to Treat Cellular Deficiencies and Dysfunction. Crit Rev Biomed Eng (2011) 39:473–91. doi: 10.1615/CritRevBiomedEng.v39.i6.10 PMC3246397 22196222

[149] Desai T Shea LD. Advances in Islet Encapsulation Technologies. Nat Rev Drug Discovery (2017) 16:338–50. doi: 10.1038/nrd.2016.232 PMC11286215 28008169

[150] Falorni A Basta G Santeusanio F Brunetti P Calafiore R. Culture Maintenance of Isolated Adult Porcine Pancreatic Islets in Three-Dimensional Gel Matrices: Morphologic and Functional Results. Pancreas (1996) 12:221–9. doi: 10.1097/00006676-199604000-00003 8830327

[151] Vegas AJ Veiseh O Gürtler M Millman JR Pagliuca FW Bader AR. Long-Term Glycemic Control Using Polymer-Encapsulated Human Stem Cell–Derived Beta Cells in Immune-Competent Mice. Nat Med (2016) 22:306–11. doi: 10.1038/nm.4030 PMC4825868 26808346

[152] Buchwald P Tamayo-Garcia A Manzoli V Tomei AA Stabler CL. Glucose-Stimulated Insulin Release: Parallel Perifusion Studies of Free and Hydrogel Encapsulated Human Pancreatic Islets. Biotechnol Bioeng (2018) 115:232–45. doi: 10.1002/bit.26442 PMC5699962 28865118

[153] Iwata H Takagi T Amemiya H Shimizu H Yamashita K Kobayashi K. Agarose for a Bioartificial Pancreas. J Biomed Mater Res (1992) 26:967–77. doi: 10.1002/jbm.820260711 1607377

[154] Iwata H Takagi T Kobayashi K Oka T Tsuji T Ito F. Strategy for Developing Microbeads Applicable to Islet Xenotransplantation Into a Spotaneous Diabetic NOD Mouse. J Biomed Mater Res (1994) 28:1201–7. doi: 10.1002/jbm.820281010 7829549

[155] Tun T Inoue K Hayashi H Aung T Gu Y-J Doia R. A Newly Developed Three-Layer Agarose Microcapsule for a Promising Biohybrid Artificial Pancreas: Rat to Mouse Xenotransplantation. Cell Transpl (1996) 5:S59–63. doi: 10.1016/0963-6897(96)00042-5 8889234

[156] Patel SN Ishahak M Chaimov D Velraj A LaShoto D Buchwald P. Organoid Microphysiological System Preserves Pancreatic Islet Function Within 3D Matrix. Sci Adv (2021) 7:eaba5515. doi: 10.1126/sciadv.aba5515 33579705 PMC7880596

[157] Lin CC Anseth KS. Glucagon-Like Peptide-1 Functionalized PEG Hydrogels Promote Survival and Function of Encapsulated Pancreatic β-Cells. Biomacromolecules (2009) 10:2460–7. doi: 10.1021/bm900420f PMC2745231 19586041

[158] Kloxin AM Kloxin CJ Bowman CN Anseth KS. Mechanical Properties of Cellularly Responsive Hydrogels and Their Experimental Determination. Adv Mater (2010) 22:3484–94. doi: 10.1002/adma.200904179 PMC3890982 20473984

[159] Weber LM Anseth KS. Hydrogel Encapsulation Environments Functionalized With Extracellular Matrix Interactions Increase Islet Insulin Secretion. Matrix Biol (2008) 27:667–73. doi: 10.1016/j.matbio.2008.08.001 PMC2631362 18773957

[160] Weaver JD Headen DM Hunckler MD Coronel MM Stabler CL García AJ. Design of a Vascularized Synthetic Poly(Ethylene Glycol) Macroencapsulation Device for Islet Transplantation. Biomaterials (2018) 172:54–65. doi: 10.1016/j.biomaterials.2018.04.047 29715595 PMC5967258

[161] Llacua LA de Haan BJ de Vos P. Laminin and Collagen IV Inclusion in Immunoisolating Microcapsules Reduces Cytokine-Mediated Cell Death in Human Pancreatic Islets. J Tissue Eng Regen Med (2018) 12:460–7. doi: 10.1002/term.2472 28508555

[162] Crisóstomo J Pereira AM Bidarra SJ Gonçalves AC Granja PL Coelho JFJ. ECM-Enriched Alginate Hydrogels for Bioartificial Pancreas: An Ideal Niche to Improve Insulin Secretion and Diabetic Glucose Profile. J Appl Biomater Funct Mater (2019) 17(4). doi: 10.1177/2280800019848923 31623515

[163] Lee BR. *Et al. In Situ* Formation and Collagen-Alginate Composite Encapsulation of Pancreatic Islet Spheroids. Biomaterials (2012) 33:837–45. doi: 10.1016/j.biomaterials.2011.10.014 22054535

[164] Nagata N Iwanaga A Inoue K Tabata Y. Co-Culture of Extracellular Matrix Suppresses the Cell Death of Rat Pancreatic Islets. J Biomater Sci Polym Ed (2002) 13:579–90. doi: 10.1163/15685620260178418 12182560

[165] Llacua A De Haan BJ Smink SA De Vos P. Extracellular Matrix Components Supporting Human Islet Function in Alginate-Based Immunoprotective Microcapsules for Treatment of Diabetes. J Biomed Mater Res - Part A (2016) 104:1788–96. doi: 10.1002/jbm.a.35706 26990360

[166] Zbinden A. Collagen and Endothelial Cell Coculture Improves β-Cell Functionality and Rescues Pancreatic Extracellular Matrix (2021). Available at: https://home.liebertpub.com/tea. doi: 10.1089/ten.tea.2020.0250 33023407

[167] Pozzi A Yurchenco PD Iozzo RV. The Nature and Biology of Basement Membranes. Matrix Biol (2017) 57–58:1–11. doi: 10.1016/j.matbio.2016.12.009 PMC5387862 28040522

[168] Kuehn C Lakey JRT Lamb MW Vermette P. Young Porcine Endocrine Pancreatic Islets Cultured in Fibrin Show Improved Resistance Toward Hydrogen Peroxide. Islets (2013) 5:207–15. doi: 10.4161/isl.26989 PMC4010573 24262980

[169] Kuehn C Fülöp T Lakey JRT Vermette P. Young Porcine Endocrine Pancreatic Islets Cultured in Fibrin and Alginate Gels Show Improved Resistance Towards Human Monocytes. Pathol Biol (2014) 62:354–64. doi: 10.1016/j.patbio.2014.07.010 25239278

[170] Andrades P Asiedu C Rodriguez C Goodwin J Deckard LA Jargal U. Insulin Secretion From Pancreatic Islets in Fibrin Glue Clots at Different Fibrinogen and Thrombin Concentrations. Transpl Proc (2007) 39:1607–8. doi: 10.1016/j.transproceed.2007.01.078 17580199

[171] Najjar M Manzoli V Abreu M Villa C Martino MM Molano RD. Fibrin Gels Engineered With Pro-Angiogenic Growth Factors Promote Engraftment of Pancreatic Islets in Extrahepatic Sites in Mice. Biotechnol Bioeng (2015) 112:1916–26. doi: 10.1002/bit.25589 25786390

[172] Moya ML Hsu Y-H Lee AP Hughes CCW George SC. *In Vitro* Perfused Human Capillary Networks. Tissue Eng Part C Methods (2013) 19:730–7. doi: 10.1089/ten.tec.2012.0430 PMC3719485 23320912

[173] Bender RHF O'Donnell BT Shergill B Pham BQ Juat DJ Hatch MS. A Vascularized 3D Model of the Human Pancreatic Islet for Ex Vivo Study of Immune Cell-Islet Interaction. bioRxiv (2021):2021.12.21.473744. doi: 10.1101/2021.12.21.473744

[174] Harrington S Williams J Rawal S Ramachandran K Stehno-Bittel L. Hyaluronic Acid/Collagen Hydrogel as an Alternative to Alginate for Long-Term Immunoprotected Islet Transplantation. Tissue Eng - Part A (2017) 23:1088–99. doi: 10.1089/ten.tea.2016.0477 PMC6112162 28142500

[175] Giobbe GG Crowley C Luni C Campinoti S Khedr M Kretzschmar K. Extracellular Matrix Hydrogel Derived From Decellularized Tissues Enables Endodermal Organoid Culture. Nat Commun (2019) 10:1–14. doi: 10.1038/s41467-019-13605-4 31827102 PMC6906306

[176] Saldin LT Cramer MC Velankar SS White LJ Badylak SF. Extracellular Matrix Hydrogels From Decellularized Tissues: Structure and Function. Acta Biomaterialia (2017) 49:1–15. doi: 10.1016/j.actbio.2016.11.068 27915024 PMC5253110

[177] Guruswamy Damodaran R Vermette P. Decellularized Pancreas as a Native Extracellular Matrix Scaffold for Pancreatic Islet Seeding and Culture. J Tissue Eng Regen Med (2018) 12:1230–7. doi: 10.1002/term.2655 29499099

[178] Mirmalek-Sani SH Orlando G McQuilling JP Pareta R Mack DL Salvatori M. Porcine Pancreas Extracellular Matrix as a Platform for Endocrine Pancreas Bioengineering. Biomaterials (2013) 34:5488–95. doi: 10.1016/j.biomaterials.2013.03.054 PMC3680884 23583038

[179] Gaetani R Aude S DeMaddalena LL Strassle H Dzieciatkowska M Wortham M. Evaluation of Different Decellularization Protocols on the Generation of Pancreas-Derived Hydrogels. Tissue Eng - Part C Methods (2018) 24:697–708. doi: 10.1089/ten.tec.2018.0180 30398401 PMC6306687

[180] Bi H Karanth SS Ye K Stein R Jin S. Decellularized Tissue Matrix Enhances Self-Assembly of Islet Organoids From Pluripotent Stem Cell Differentiation. ACS Biomater Sci Eng (2020) 6:4155–65. doi: 10.1021/acsbiomaterials.0c00088 33463310

[181] Narayanan K Lim VY Shen J Tan ZW Rajendran D Luo S-C. Extracellular Matrix-Mediated Differentiation of Human Embryonic Stem Cells: Differentiation to Insulin-Secreting Beta Cells. Tissue Eng Part A (2013) 20:424–33. doi: 10.1089/ten.tea.2013.0257 24020641

[182] Bi H Ye K Jin S. Proteomic Analysis of Decellularized Pancreatic Matrix Identifies Collagen V as a Critical Regulator for Islet Organogenesis From Human Pluripotent Stem Cells. Biomaterials (2020) 233:119673. doi: 10.1016/j.biomaterials.2019.119673 31866049

[183] Pouliot RA Link PA Mikhaiel NS Schneck MB Valentine MS Gninzeko FJK. Development and Characterization of a Naturally Derived Lung Extracellular Matrix Hydrogel. J Biomed Mater Res Part A (2016) 104:1922–35. doi: 10.1002/jbm.a.35726 PMC7962169 27012815

[184] Buchwald P. FEM-Based Oxygen Consumption and Cell Viability Models for Avascular Pancreatic Islets. Theor Biol Med Model (2009) 6:1–13. doi: 10.1186/1742-4682-6-5 19371422 PMC2678100

[185] Low LA Mummery C Berridge BR Austin CP Tagle DA. Organs-On-Chips: Into the Next Decade. Nat Rev Drug Discovery (2021) 20:345–61. doi: 10.1038/s41573-020-0079-3 32913334

[186] The University of Pittsburgh Drug Discovery Institute Microphysiology Database Project. (2021). Available at: https://mps.csb.pitt.edu/microdevices/model/ (Accessed 3rd June 2021).

[187] Santini-González J Simonovich JA Castro-Gutiérrez R González-Vargasac Y Abuid NJ Stabler CL. *In Vitro* Assembly of Peri-Islet Basement Membrane-Like Structures. Biomaterials (2021) 273:120808. doi: 10.1016/j.biomaterials.2021.120808 33895491 PMC8131001

[188] Homan KA Gupta N Kroll KT Kolesky DB Skylar-Scott M Miyoshi T. Flow-Enhanced Vascularization and Maturation of Kidney Organoids *In Vitro* . Nat Methods (2019) 16:255–62. doi: 10.1038/s41592-019-0325-y PMC6488032 30742039

[189] Bandak B Yi L Roper MG. Microfluidic-Enabled Quantitative Measurements of Insulin Release Dynamics From Single Islets of Langerhans in Response to 5-Palmitic Acid Hydroxy Stearic Acid. Lab Chip (2018) 18:2873–82. doi: 10.1039/C8LC00624E PMC6133761 30109329

[190] Yi L Wang X Dhumpa R Schrell AM Mukhitov N Roper MG. Integrated Perfusion and Separation Systems for Entrainment of Insulin Secretion From Islets of Langerhans. Lab Chip (2015) 15:823–32. doi: 10.1039/C4LC01360C PMC4304979 25474044

[191] Dishinger JF Kennedy RT. Serial Immunoassays in Parallel on a Microfluidic Chip for Monitoring Hormone Secretion From Living Cells. Anal Chem (2007) 79:947–54. doi: 10.1021/ac061425s 17263320

[192] Godwin LA Pilkerton ME Deal KS Wanders D Judd RL Easley CJ. Passively Operated Microfluidic Device for Stimulation and Secretion Sampling of Single Pancreatic Islets. Anal Chem (2011) 83:7166–72. doi: 10.1021/ac201598b PMC4980096 21806019

[193] Lu S Dugan CE Kennedy RT. Microfluidic Chip With Integrated Electrophoretic Immunoassay for Investigating Cell-Cell Interactions. Anal Chem (2018) 90:5171–8. doi: 10.1021/acs.analchem.7b05304 PMC6943824 29578696

[194] Chen D Pilkerton ME Deal KS Wanders D Judd RL Easley CJ. The Chemistrode: A Droplet-Based Microfluidic Device for Stimulation and Recording With High Temporal, Spatial, and Chemical Resolution. Proc Natl Acad Sci USA (2008) 105:16843–8. doi: 10.1073/pnas.0807916105 PMC2579341 18974218

[195] Chen W Lisowski M Khalil G Sweet IR Shen AQ. Microencapsulated 3-Dimensional Sensor for the Measurement of Oxygen in Single Isolated Pancreatic Islets. PLoS One (2012) 7:e33070. doi: 10.1371/journal.pone.0033070 22479359 PMC3315556

[196] Sankar KS Green BJ Crocker AR Verity JE Altamentova SM Rocheleau JV. Culturing Pancreatic Islets in Microfluidic Flow Enhances Morphology of the Associated Endothelial Cells. PLoS One (2011) 6:24904. doi: 10.1371/journal.pone.0024904 PMC3178551 21961048

[197] Lee SH Hong SG Song J Cho B Han EJ Kondapavulur S. Microphysiological Analysis Platform of Pancreatic Islet β-Cell Spheroids. Adv Healthc Mater (2018) 7:1701111. doi: 10.1002/adhm.201701111 29283208

[198] Wang Y Lee D Zhang L Jeon H Mendoza-Elias JE Harvat TA. Systematic Prevention of Bubble Formation and Accumulation for Long-Term Culture of Pancreatic Islet Cells in Microfluidic Device. Biomed Microdevices (2012) 14:419–26. doi: 10.1007/s10544-011-9618-3 PMC3303988 22252566

[199] Chen YY Silva PN Syed AM Sindhwani S Rocheleau JV Chan WCW. Clarifying Intact 3D Tissues on a Microfluidic Chip for High-Throughput Structural Analysis. Proc Natl Acad Sci USA (2016) 113:14915–20. doi: 10.1073/pnas.1609569114 PMC5206515 27956625

[200] Glieberman AL Pope BD Zimmerman JF Liu Q Ferrier JP Jr. Kenty JHR. Synchronized Stimulation and Continuous Insulin Sensing in a Microfluidic Human Islet on a Chip Designed for Scalable Manufacturing. Lab Chip (2019) 19:2993–3010. doi: 10.1039/C9LC00253G 31464325 PMC6814249

[201] Heileman K Daoud J Hasilo C Gasparrini M Paraskevas S Tabrizian M. Microfluidic Platform for Assessing Pancreatic Islet Functionality Through Dielectric Spectroscopy. Biomicrofluidics (2015) 9:44125. doi: 10.1063/1.4929652 PMC4552695 26339324

[202] Xing Y Nourmohammadzadeh M Elias JEM Chan M Chen Z McGarrigle JJ. A Pumpless Microfluidic Device Driven by Surface Tension for Pancreatic Islet Analysis. Biomed Microdevices (2016) 18:1–9. doi: 10.1007/s10544-016-0109-4 27534648

[203] Miyazaki J-I Araki K Yamato E Ikegami H Asano T Shibasaki Y. Establishment of a Pancreatic β Cell Line That Retains Glucose-Inducible Insulin Secretion: Special Reference to Expression of Glucose Transporter Isoforms*. Endocrinology (1990) 127:126–32. doi: 10.1210/endo-127-1-126 2163307

[204] Lo JF Wang Y Blake A Yu G Harvat TA Jeon H. Islet Preconditioning *via* Multimodal Microfluidic Modulation of Intermittent Hypoxia. Anal Chem (2012) 84:1987–93. doi: 10.1021/ac2030909 PMC3302975 22296179

[205] Lenguito G Chaimov D Weitz JR Rodriguez-Diaz R Rawal SAK Tamayo-Garcia A. Resealable, Optically Accessible, PDMS-Free Fluidic Platform for *Ex Vivo* Interrogation of Pancreatic Islets. Lab Chip (2017) 17:772–81. doi: 10.1039/C6LC01504B PMC5330806 28157238

[206] Bauer S Huldt CW Kanebratt KP Durieux I Gunne D Andersson S. Functional Coupling of Human Pancreatic Islets and Liver Spheroids on-a-Chip: Towards a Novel Human *Ex Vivo* Type 2 Diabetes Model. Sci Rep (2017) 7:1–11. doi: 10.1038/s41598-017-14815-w 29097671 PMC5668271

[207] Lee D Wang Y Mendoza-Elias JE Adewola AF Harvat TA Kinzer K. Dual Microfluidic Perifusion Networks for Concurrent Islet Perifusion and Optical Imaging. Biomed Microdevices (2012) 14:7–16. doi: 10.1007/s10544-011-9580-0 21850483 PMC3696955

[208] Ishahak M Hill J Amin Q Wubker L Hernandez A Mitrofanova A. Modular Microphysiological System for Modeling of Biologic Barrier Function. Front Bioeng Biotechnol (2020) 8:581163. doi: 10.3389/fbioe.2020.581163 33304889 PMC7693638

[209] Li X Brooks JC Hu J Ford KI Easley CJ. 3D-Templated, Fully Automated Microfluidic Input/Output Multiplexer for Endocrine Tissue Culture and Secretion Sampling. Lab Chip (2017) 17:341–9. doi: 10.1039/C6LC01201A PMC5293597 27990542

[210] Mohammed JS Wang Y Harvat TA Oberholzer J Eddington DT. Microfluidic Device for Multimodal Characterization of Pancreatic Islets. Lab Chip (2009) 9:97–106. doi: 10.1039/B809590F 19209341 PMC3759253

[211] Adewola AF Lee J Choi S Yang JH Sander M Chung S. Microfluidic Perifusion and Imaging Device for Multi-Parametric Islet Function Assessment. Biomed Microdevices (2010) 12:409–17. doi: 10.1007/s10544-010-9398-1 20300858

[212] Jun Y. *Et al. In Vivo*–Mimicking Microfluidic Perfusion Culture of Pancreatic Islet Spheroids. Sci Adv (2019) 5:4520–47. doi: 10.1126/sciadv.aax4520 PMC6881167 31807701

[213] Huh D Kim HJ Fraser JP Shea DE Khan M Bahinski A. Microfabrication of Human Organs-on-Chips. Nat Protoc (2013) 8:2135–57. doi: 10.1038/nprot.2013.137 24113786

[214] McDonald JC Whitesides GM. Poly(dimethylsiloxane) as a Material for Fabricating Microfluidic Devices. Acc Chem Res (2002) 35:491–9. doi: 10.1021/ar010110q 12118988

[215] Berthier E Young EWK Beebe D. Engineers are From PDMS-Land, Biologists are From Polystyrenia. Lab Chip (2012) 12:1224–37. doi: 10.1039/c2lc20982a 22318426

[216] van Meer BJ de Vries H Firth KSA van Weerd J Tertoolen LGJ Karperien HBJ. Small Molecule Absorption by PDMS in the Context of Drug Response Bioassays. Biochem Biophys Res Commun (2017) 482:323–8. doi: 10.1016/j.bbrc.2016.11.062 PMC5240851 27856254

[217] Adiraj Iyer M Eddington DT. Storing and Releasing Rhodamine as a Model Hydrophobic Compound in Polydimethylsiloxane Microfluidic Devices. Lab Chip (2019) 19:574–9. doi: 10.1039/C9LC00039A 30681692

[218] Nunemaker CS Dishinger JF Dula SB Wu R Merrins MJ Reid KR. Glucose Metabolism, Islet Architecture, and Genetic Homogeneity in Imprinting of [Ca2+]i and Insulin Rhythms in Mouse Islets. PLoS One (2009) 4:8428. doi: 10.1371/journal.pone.0008428 PMC2793028 20037650

[219] Glieberman AL Pope BD Melton DA Parker KK. Building Biomimetic Potency Tests for Islet Transplantation. Diabetes (2021) 70:347–63. doi: 10.2337/db20-0297 PMC7881865 33472944

[220] Shackman JG Dahlgren GM Peters JL Kennedy RT. Perfusion and Chemical Monitoring of Living Cells on a Microfluidic Chip. Lab Chip (2005) 5:56–63. doi: 10.1039/b404974h 15616741

[221] Lomasney AR Yi L Roper MG. Simultaneous Monitoring of Insulin and Islet Amyloid Polypeptide Secretion From Islets of Langerhans on a Microfluidic Device. Anal Chem (2013) 85:7919–25. doi: 10.1021/ac401625g PMC3770151 23848226

[222] Guillo C Roper MG. Simultaneous Capillary Electrophoresis Competitive Immunoassay for Insulin, Glucagon, and Islet Amyloid Polypeptide Secretion From Mouse Islets of Langerhans. J Chromatogr A (2011) 1218:4059–64. doi: 10.1016/j.chroma.2011.05.006 PMC3109176 21620410

[223] Takahashi A Camacho P Lechleiter JD Herman B. Measurement of Intracellular Calcium. Physiol Rev (1999) 79:1089–125. doi: 10.1152/physrev.1999.79.4.1089 10508230

[224] Almaça J Molina J Menegaz D Pronin AN Tamayo A Slepak V. Human Beta Cells Produce and Release Serotonin to Inhibit Glucagon Secretion From Alpha Cells. Cell Rep (2016) 17:3281–91. doi: 10.1016/j.celrep.2016.11.072 PMC5217294 28009296

[225] Menegaz D Hagan DW Almaça J Cianciaruso C Rodriguez-Diaz R Molina J. Mechanism and Effects of Pulsatile GABA Secretion From Cytosolic Pools in the Human Beta Cell. Nat Metab (2019) 1:1110–26. doi: 10.1038/s42255-019-0135-7 PMC7236889 32432213

[226] Marvin JS Borghuis BG Tian L Cichon J Harnett MT Akerboom J. An Optimized Fluorescent Probe for Visualizing Glutamate Neurotransmission. Nat Methods (2013) 10:162–70. doi: 10.1038/nmeth.2333 PMC4469972 23314171

[227] Rodriguez-Diaz R Dando R Huang YA Berggren P-O Roper SD Caicedo A. Real-Time Detection of Acetylcholine Release From the Human Endocrine Pancreas. Nat Protoc (2012) 7:1015–23. doi: 10.1038/nprot.2012.040 PMC3538842 22555241

[228] Reissaus CA Piñeros AR Twigg AN Orr KS Conteh AM Martinez MM. A Versatile, Portable Intravital Microscopy Platform for Studying Beta-Cell Biology *In Vivo* . Sci Rep (2019) 9:1–11. doi: 10.1038/s41598-019-44777-0 31186447 PMC6559992

[229] Vigneshvar S Sudhakumari CC Senthilkumaran B Prakash H. Recent Advances in Biosensor Technology for Potential Applications - an Overview. Front Bioeng Biotechnol (2016) 4:11. doi: 10.3389/fbioe.2016.00011 26909346 PMC4754454

[230] Liu Q Wu C Cai H Hu N Zhou J Wang P. Cell-Based Biosensors and Their Application in Biomedicine. Chem Rev (2014) 114:6423–61. doi: 10.1021/cr2003129 24905074

[231] Walker JT Saunders DC Brissova M Powers AC. The Human Islet: Mini-Organ With Mega-Impact. Endocr Rev (2021) 42(5):605–57. doi: 10.1210/endrev/bnab010 PMC8476939 33844836

[232] Castiello FR Heileman K Tabrizian M. Microfluidic Perfusion Systems for Secretion Fingerprint Analysis of Pancreatic Islets: Applications, Challenges and Opportunities. Lab Chip (2016) 16:409–31. doi: 10.1039/C5LC01046B 26732665

[233] Wasson EM Dubbin K Moya ML. Go With the Flow: Modeling Unique Biological Flows in Engineered *In Vitro* Platforms. Lab Chip (2021) 21:2095–120. doi: 10.1039/D1LC00014D 34008661

[234] Rondas D Tomas A Soto-Ribeiro M Wehrle-Haller B Halban PA. Novel Mechanistic Link Between Focal Adhesion Remodeling and Glucose-Stimulated Insulin Secretion. J Biol Chem (2012) 287:2423. doi: 10.1074/jbc.M111.279885 22139838 PMC3268403

[235] Silva PN Green BJ Altamentova SM Rocheleau JV. A Microfluidic Device Designed to Induce Media Flow Throughout Pancreatic Islets While Limiting Shear-Induced Damage. Lab Chip (2013) 13:4374–84. doi: 10.1039/c3lc50680k 24056576

